# Anti-Amyloid Aggregation Effects of Gobaishi (*Galla chinensis*) and Its Active Constituents

**DOI:** 10.3390/molecules30132720

**Published:** 2025-06-24

**Authors:** Sharmin Akter, Takayuki Tohge, Sahithya Hulimane Ananda, Masahiro Kuragano, Kiyotaka Tokuraku, Koji Uwai

**Affiliations:** 1 Laboratory of Organic Chemistry in Life Science, Muroran Institute of Technology, Muroran 050-8585, Japan; 22096504@muroran-it.ac.jp; 2 Laboratory of Plant Secondary Metabolism, Nara Institute of Science and Technology, Nara 630-0192, Japan; tohge@bs.naist.jp; 3 Laboratory of Protein Chemistry, Muroran Institute of Technology, Muroran 050-8585, Japan; 22096502@muroran-it.ac.jp (S.H.A.); gano@muroran-it.ac.jp (M.K.); tokuraku@muroran-it.ac.jp (K.T.)

**Keywords:** Gobaishi, pentagalloyl glucose, methyl gallate, amyloid-beta, antioxidant

## Abstract

Alzheimer′s disease (AD) is a chronic neurodegenerative disorder that leads to memory loss and changes in mental and behavioral functions in elderly individuals. A major pathological feature of AD is the aggregation of amyloid-beta (Aβ) peptides, along with oxidative stress, inducing neurocellular apoptosis in the brain. Gobaishi (*Galla chinensis*), a traditional herbal medicine, has gained considerable attention for its constituents and potent therapeutic properties, particularly its strong inhibitory activity against Aβ fibril formation. In this study, we investigated the anti-Aβ aggregation effects of Gobaishi and its active constituents. We isolated two compounds by employing Thioflavin T (ThT) assay-guided fractionation, which were identified through various spectroscopic methods as pentagalloyl glucose (PGG) and methyl gallate (MG). Evaluation of their anti-Aβ aggregation effects revealed that PGG and MG contribute 1.5% and 0.7% of the activity of Gobaishi, respectively. In addition, PGG demonstrated significantly stronger DPPH radical scavenging activity (EC_50_ = 1.16 µM) compared to MG (EC_50_ = 6.44 µM). At a concentration of 30 µM, PGG significantly reduced the Aβ-induced cytotoxicity in SH-SY5Y cell lines compared to MG. Based on these findings, both Gobaishi and its active compound PGG are proposed as promising candidates for further investigation as potent anti-amyloidogenic agents in AD management.

## 1. Introduction

AD is a complex and multifactorial neurodegenerative disorder that affects millions of individuals globally as the population ages. Clinically, AD is characterized by significant impairments in memory and cognitive function, behavioral instability, deficits in judgment and reasoning, and difficulties in verbal communication and language comprehension [1]. The global prevalence of AD and other dementias has been estimated to exceed 55 million, with nearly 10 million new diagnoses annually, exacerbating the burden of this condition [2,3]. The etiology of AD is likely the consequence of an intricate relationship between genetic susceptibility and environmental influences. The neuropathological hallmark of AD includes the extracellular accumulation of neurotoxic Aβ and the aggregation of microtubule-associated protein tau within the brain, leading to the formation of senile plaques that progressively drive cognitive decline and memory impairment [4]. The genetic liability associated with AD is primarily triggered by mutations in the APP, PSEN1, and PSEN2 genes, which contribute to the morphological changes associated with Aβ plaque formation by facilitating the synthesis of Aβ monomer, oligomer, protofibrils, and fibrillar deposits [5].

Additionally, several factors playing decisive roles in the pathophysiology of AD include degeneration of cholinergic neurons, hormonal imbalance, oxidative stress, defective mitochondrial function, and metal stress [6,7]. Due to the complex and multifaceted nature of AD, identifying and developing efficient therapeutics remains a considerable challenge. At present, the available therapies primarily aim to alleviate symptoms [8]. Considering Aβ as the primary hallmark of AD, there is growing interest in evaluating natural molecules inhibitory activity against Aβ aggregation, as supported by several studies [9,10].

Gobaishi, scientifically known as *Galla chinensis*, commonly referred to as Japanese gall or Chinese sumac gall, is a unique and distinctive outgrowth that develops on the *Rhus chinensis* plant leaves, which belong to the Anacardiaceae family [11]. This phenomenon occurs in response to an infestation by the white aphid *Schlechtendalia chinensis* [12,13]. These galls are significantly recognized for their medicinal value, primarily due to their unique composition, which predominantly comprises gallotannins (60–70%), gallic acid (20%), methyl gallate (7%), and an array of other phytochemicals [14,15]. This plant species is endemic to East Asia, particularly China and Japan, and is also found in tropical and subtropical regions of India, Malaysia, and Korea [16]. Previous investigations have highlighted the extensive medicinal properties of Gobaishi, attributed to its valuable chemical composition, and it has been utilized for over 2000 years to treat various ailments [17,18,19,20,21].

Previous studies have estimated that Gobaishi extracts can improve cognitive impairment in both in vivo and in vitro models, suggesting it may have neuroprotective potential for further investigation in managing AD [21,22,23]. However, to date, no studies have specifically identified or characterized the anti-amyloidogenic components of Gobaishi. Given the central role of Aβ aggregation in AD progression, this study aims to identify the compounds responsible for Gobaishi′s anti-amyloidogenic activity.

This study examines the potential inhibitory effects of the EtOH extract of Gobaishi on Aβ aggregation using a ThT assay, a widely recognized method for detecting Aβ fibrils [24]. Subsequently, we seek to identify the bioactive components in Gobaishi inhibiting Aβ aggregation through a bioassay-guided isolation process employing diverse chromatography techniques. We elucidate the molecular structures of the isolated compounds via nuclear magnetic resonance (NMR) spectroscopy and mass spectrometry. Additionally, we assess their antioxidant and cytotoxic properties to evaluate their potential therapeutic applications.

## 2. Results

### 2.1. Identification and Characterization of Major Compounds in Gobaishi

**Figure 1:**
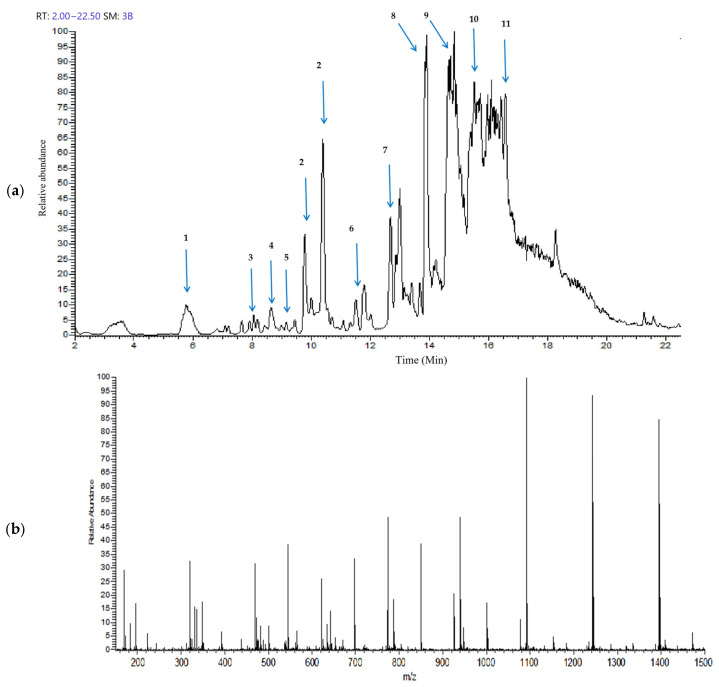
Metabolite profiling of Gobaishi using full scan mode covering negative (sid = 5) ion detection. (**a**) Total ion chromatogram of the EtOH fraction of Gobaishi. (**b**) Mass spectrum of the EtOH fraction of Gobaishi.

The Gobaishi EtOH extract was analyzed by UHPLC–ion trap MS spectrometry. Figure 1a,b represent the total ion chromatogram and the corresponding mass spectrum demonstrating a diverse range of metabolites in the EtOH extract of Gobaishi. The target compounds were identified by analyzing the retention times and their corresponding mass spectra and comparing the observed fragmentation patterns with previously reported data [14,15,20] and entries in the PubChem database.

Table 1 represents the main molecular mass ranges, corresponding compounds, and *m*/*z* values observed in the Gobaishi EtOH extract from the negative (sid = 5) ion MS spectrometry experiments. This extract is mainly composed of gallic acids and its derivative, and a variety of galloyl esters of glucose.

### 2.2. Isolation of Aβ Aggregation Inhibitors from Gobaishi

**Figure 2:**
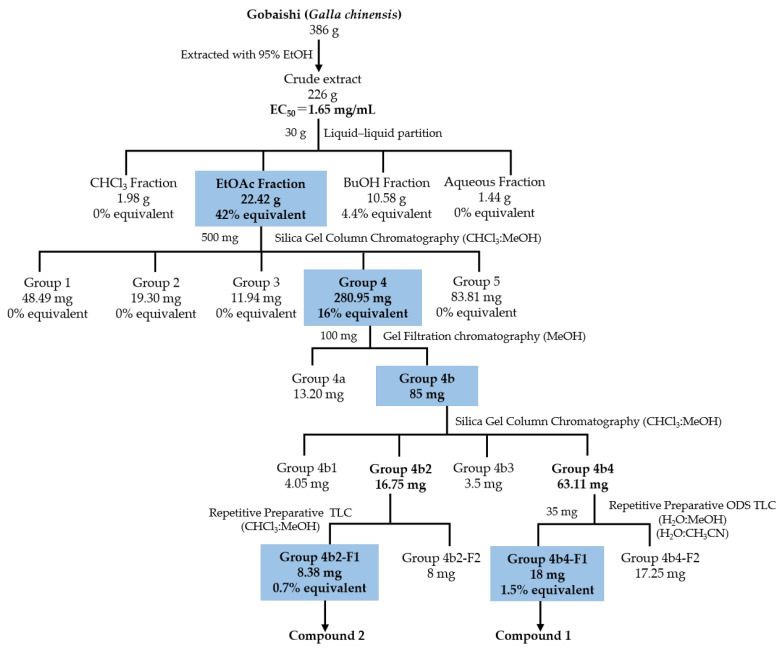
Isolation of Aβ aggregation inhibitor derived from Gobaishi.

Figure 2 illustrates the bioassay-guided isolation of Aβ aggregation inhibitor from the Gobaishi extract. Initial evaluation using a ThT assay showed that Gobaishi crude extract exhibited strong inhibitory effect against Aβ42 aggregation (EC_50_ = 1.65 mg/mL). Subsequently, the crude extract was partitioned into four distinct fractions: CHCl_3_, EtOAc, BuOH, and water. Each fraction was evaluated for its inhibitory activity on Aβ42 aggregation based on its yield concentration. The results indicated that the EtOAc and BuOH fractions contribute 42% and 4.4% of the overall activity of Gobaishi, respectively (Figure 2 and Table 2).

Based on the initial ThT results for these four fractions, the Gobaishi EtOAc fraction was selected for further bioassay-guided isolation. This fraction was subjected to open-column chromatography, ODS column chromatography, and gel filtration chromatography using Sephadex LH-20. As a result, two compounds (compound **1** and **2**) were successfully isolated (Figure 2).

### 2.3. Structure Elucidation of the Active Compounds

**Figure 3:**
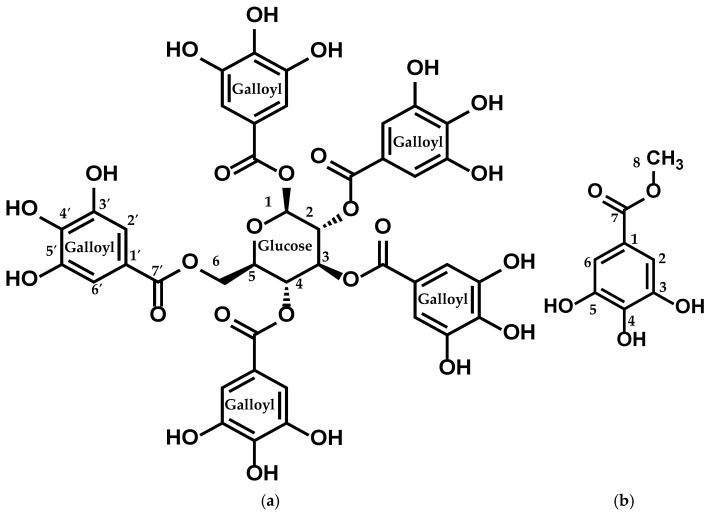
Structure of (**a**) compound **1** (PGG) and (**b**) compound **2** (MG).

The structures of the isolated compounds were elucidated by performing ^1^H, ^13^C, DEPT, HMQC, and HMBC NMR, as depicted in Figure 3 and detailed in Table 3 and Table 4.

Compound **1**: This compound showed a molecular formula of C_41_H_32_O_26_, evidenced by a molecular-adduct ion peak at *m*/*z* 963 [M + Na]^+^ observed in the LC-MS/MS (positive mode) spectrum.

The ^1^H-NMR spectrum of the isolated compound showed five aromatic singlets, each integrating for 2 protons at δ 7.11 (s, 2H), 7.05 (s, 2H), 6.98 (s, 2H), 6.95 (s, 2H), and 6.90 (s, 2H), indicating the presence of multiple substituted aromatic protons. Additional signals observed at δ 6.24 (d, *J* = 8.6 Hz, 1H), 5.91 (t, *J* = 9.7 Hz, 1H), 5.64–5.57 (m, 2H), 4.51 (d, *J* = 10.3 Hz, 1H), and 4.43–4.36 (m, 2H) were attributed to oxymethine and oxymethelene protons. The connectivity of these protons was observed by the ^1^H-^1^H COSY and HMBC spectra.

The ^13^C-NMR spectrum of isolated compound **1** exhibited six carbon signals in the range of δ (93.8–63.1), attributed to the oxymethine and oxymethelene carbons. The five-carbon signals at δ 167.9, 167.3, 167.0, 166.9, and 166.2 indicated aromatic carboxyl derivatives.

The cross-peaks of the signal at δ 167.9 (C-7′) with the signals at δ 7.11 (H-2′ and H-6′) and δ 6.24 (H-1), characterized by the HMQC and HMBC spectra, show the interconnection between the aromatic signals and the oxymethine signals. In addition, PGG was identified by comparing the spectroscopic data reported in the literature [25,26].

Compound **2**: This compound showed a molecular formula of C_8_H_8_O_5_, evidenced by a [M + H]^+^ ion peak at *m*/*z* 185 observed in the LC-MS/MS (positive mode) spectrum. ^1^H-NMR spectra of the isolated compound displayed the presence of an aromatic singlet, attributing two protons at δ 6.99 (s, 2H). The broad singlet at δ 3.74 indicates the presence of three protons, indicating the oxymethyl protons.

The four carbon signals at around δ 144.6, 138.1, 120.8, and 109.9 represent the substituted aromatic ring, and the carbon signal at δ 169.0 (C7) indicates the presence of a carboxyl derivative. Finally, the interrelation between the aromatic ring and oxymethyl protons was characterized by the HMBC and DEPT-135 spectra, showing that the signals at δ 7.0 (H-2 and H-6) and δ 3.74 (H-1) were interlinked by the carbon signal at δ 169 (C-7′). Finally, MG was identified by comparing the spectroscopic data described in the literature [27,28] (Figure 3).

### 2.4. Aβ42 Aggregation Inhibitory Activity Monitored by ThT Assay

Thioflavin T (ThT) is a small aromatic compound that exhibits significant fluorescence upon binding to amyloid fibrils, enabling the quantification of amyloid fibrils [29]. In this study, the ThT assay was carried out to elucidate the inhibitory effects on amyloid fibril formation following a 24 h incubation at 37 °C with extracts from Gobaishi, its subfractions, and the isolated compounds. Rosmarinic acid was utilized as a reference compound (EC_50_ = 15 µM), presented in Supplementary Figure S19. The bioactivity contribution of EtOAc fraction was about 42% of the inhibitory potential of the Gobaishi EtOH extract. The isolated compound **1** and compound **2** from the EtOAc extract were calculated to account for 1.5% and 0.7% of the bioactive potential of the EtOH extract of Gobaishi, respectively (Figure 2).

**Figure 4:**
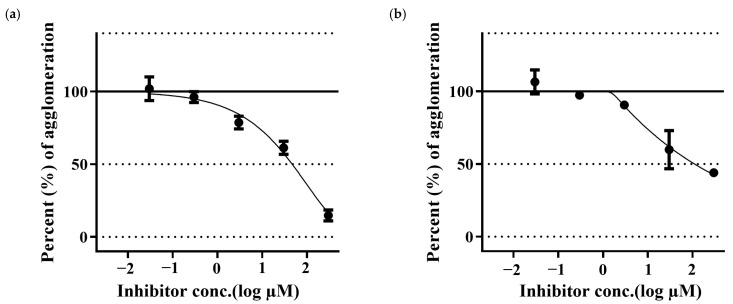
Inhibition of Aβ42 aggregation by pentagalloyl glucose (**a**) and methyl gallate (**b**). The data represent inhibitory activity as % of Aβ aggregation and are expressed as the mean ± SD; *n* = 3.

Aβ42 samples treated with PGG have shown a reduction in the Aβ fibrillation in a dose-dependent manner. PGG has shown stronger inhibitory activity than that of MG. (Figure 4).

### 2.5. Confirmation of Aβ Aggregate Formation

**Figure 5:**
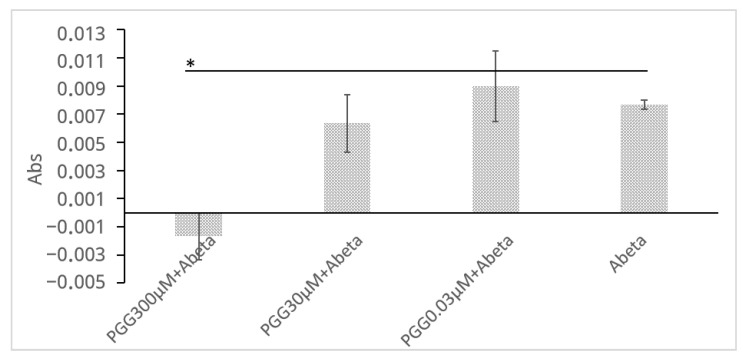
PGG inhibits Aβ aggregation as assessed by a turbidity assay. The legends are obtained from the Abs of (PGG + Aβ)−PGG. Data are presented as the mean ± SD of Abs of three independent experiments (*n* = 3). The asterisk (*) indicates a statistically significant difference compared to the Aβ alone group (one-tailed Student′s *t*-test, * *p* < 0.05).

To complement the ThT assay and provide direct physicochemical evidence that PGG inhibits the formation of large aggregates, a turbidity assay was performed (Figure 5). The measurement was conducted at 450 nm to avoid interference from the intrinsic absorbance of PGG itself (<400 nm). Although the light-scattering signal is inherently weaker at this longer wavelength, incubation of Aβ (30 μM) alone for 24 h led to a distinct increase in solution turbidity (ΔAbs = 0.0077 ± 0.0003). In stark contrast, this turbidity increase was completely suppressed to baseline levels when Aβ was co-incubated with PGG (300 μM), and this difference was statistically significant (ΔAbs = −0.0017 ± 0.0017, *p* = 0.029). This result provides physical evidence that PGG directly inhibits the growth of large, light-scattering Aβ aggregates.

### 2.6. DPPH Radical Scavenging Activity of Isolated Compounds

The DPPH radical scavenging activity was employed to evaluate the antioxidant′s free radical scavenging capacity of PGG and MG. α-Tocopherol was used as a positive control. PGG revealed to be significantly more potent radical scavengers than that of MG and the positive control, as presented in Table 5. Both compounds showed highly significant antioxidant activity. PGG showed slightly stronger statistical difference (*p* = 9.99 × 10^−8^) than MG (*p* = 7.65 × 10^−6^) when compared to tocopherol, suggesting a potentially greater effect, though both effects were robust.

### 2.7. Inhibition of Aβ-Induced Cytotoxicity

**Figure 6:**
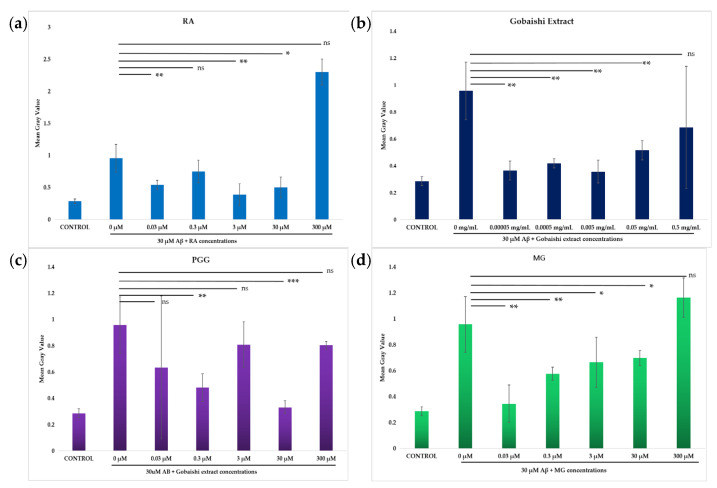
Effect of RA (**a**), Gobaishi extract (**b**), PGG (**c**), and MG (**d**) on SH-SY5Y cells in the presence or absence of 30 μM Aβ at different concentrations. Results represent the mean  ±  SD (*n*  =  3). Differences between groups (Aβ vs. treatments) were evaluated for statistical significance using two-tailed Student′s *t*-test. * *p*  <  0.05, ** *p*  <  0.005, *** *p*  <  0.0005. ns stands for not significant.

Incubation of SH-SY5Y cells with 30 μM Aβ results in significant cytotoxicity. The neuroprotective effects of the test compounds (0.03–300 μM) and extract (0.00005–0.5 mg/mL) against Aβ-induced cytotoxicity in SH-SY5Y cells were evaluated via propidium iodide (PI) uptake following treatment incubation with 30 μM Aβ. Figure 5 shows that both the extract and compounds significantly reduced the Aβ aggregation-induced cytotoxicity at different concentrations. The extract significantly reduced the Aβ-induced cell death across the range of concentrations, though the reduced activity at 0.5 mg/mL was not statistically significant (Figure 6b). The reference compound (RA) and the test compounds (PGG and MG) showed significant neuroprotection, against Aβ-induced cell death at different concentrations (Figure 6a,c,d). However, the activity of MG at the concentration of 300 μM and the activity of PGG and RA at the concentration of 300 μM and 3 μM were not significant. Among them, PGG exhibits the strongest protective effect at 30 μM, presented in Figure 6c.

## 3. Discussion

Traditional Chinese and Japanese medicine comprise a wide variety of bioactive compounds, making them significant and valuable alternatives within the global healthcare system. While several naturally derived compounds such as curcumin, pinitol, resveratrol, L-clausenamide, and their derivatives have been shown to play significant roles in reducing AD pathology by combining multiple pathways [30,31], their efficacy has not yet been fully determined. Therefore, studying medicinal plants is pivotal, as it not only directs us towards the effective bioactive compound but also has the potential to influence the modification of the lead compound to enhance their biochemical properties [32]. Our findings may provide a basis for further investigation towards the development of novel pharmaceuticals.

In this study, the major chemical composition of the Gobaishi EtOH extract was elucidated as a series of galloylglucoses. Also, the EtOH extract of Gobaishi significantly decreased the aggregation of Aβ42 fibrils. In addition, two compounds were isolated as active constituents. Among them, PGG showed potent inhibitory activity compared to MG. However, their individual effects were found to be lower than those of the Gobaishi EtOH extract as well as EtOAc extract. Furthermore, both compounds exhibited antioxidant capacity as well as reduced cytotoxic effects in SH-SY5Y cell lines, with PGG being comparatively more efficient than MG in both aspects. These results suggest that PGG may be a promising candidate for future research into its potential therapeutic applications.

Our findings about the metabolite profile of Gobaishi resonates with a previous study supporting that *Galla chinensis* comprises (mono-deca) galloyl glucoses [15], highlighting the structural specificity of those metabolites in these galls.

A limited number of prior studies have reported that Gobaishi extract exhibits the potential to enhance neurocognitive performance in AD model [22,23]. However, the composition of Gobaishi, responsible for the anti-amyloidgenic activity, remains unknown. This study investigated the anti-amyloidogenic compositions of Gobaishi. Among the multitude of contributory factors of AD, toxic Aβ aggregates play a significant role. The structural backbone of these toxic Aβ aggregates is rich in beta-sheet conformations, which possess an inclination to interact, ultimately leading to the fibrillogenesis of toxic amyloid plaques [33]. Consequently, inhibiting the formation of a beta-sheet structure may counteract the aggregation pathway, thereby impeding oligomer formation and preventing pathogenic fibril deposition. Previous studies have reported that gallotannians are synthesized through the esterification of glucose moieties with galloyl groups, resulting in a high content of phenolic -OH groups [15]. These phenolic -OH groups can form intermolecular interactions with both carbonyl and amine groups of Aβ peptides, thus disrupting the aggregate structure [34]. Researchers have emphasized the study of bioactive compound-rich medicinal plants like grape seed extract, *Ginko biloba* extract, *Glycyrrhiza glabra*, and their role as a multifunctional nutraceutical for treating neurological disorders [35,36].

PGG has previously been reported to inhibit Aβ42 fibrillation and disassemble the pre-formed Aβ42 fibrils. A study conducted on *Paenia suffruticosa* utilized concentrations of 10 µM and 20 µM of Aβ42, which are comparatively lower than the Aβ42 concentration (30 µM) used in our study [37]. Therefore, differences in Aβ42 concentrations across studies may influence the comparability of these results. Moreover, PGG was also isolated from *Terminalia chebula* fruits, *Rhodiola crenulata* roots, and *Rhus chinensis* plant, showing critical biological activities such as AChE-, BChE-, and BACE1-inhibitory effects and anti-inflammatory effects [38,39], suggesting that it can be a potential multitargeting therapeutic agent of AD. On the other hand, a previous study reported that synthesized MG can reduce of Aβ aggregation [40]. Notably, while that study employed UV-visible spectroscopy to assess the inhibitory activity on Aβ25–35, our study used the ThT assay with Aβ42. Despite the differences in methodology and Aβ fragments, our study supports the effectiveness of MG in inhibiting Aβ aggregation.

Antioxidants play a crucial role in protecting neurons from oxidative stress and Aβ aggregation, both of which are implicated in AD pathology [41,42]. Previous reports have highlighted the potential antioxidant activity of Gobaishi extract due to its rich content of gallotannins, playing an essential role in enhancing cognitive function [21,22]. In our study, both the isolated compounds exhibited potent DPPH radical scavenging activity within a concentration range of 7–125 μg/mL using 0.5 mM DPPH. A prior study reported similar radical scavenging effects with PGG at concentrations ranging from 1 to 100 μg/mL but using a lower DPPH concentration of 0.1 mM [43]. Despite the differences in DPPH concentrations, both studies demonstrate higher antioxidant activity of PGG than the standard. This consistent finding of strong radical scavenging capability across studies supports the potential of these compounds as effective antioxidants. Compounds with hydroxyl (-OH) functional groups typically exhibit antioxidant properties due to their ability to donate hydrogen atoms [44]. Such structural features support multifunctionality, though not always via the same pathway. As reported earlier, epigallocatechin possesses multiple hydroxyl groups, contributing to the Aβ aggregation inhibition by acting as an antioxidant and by regulating inflammation [45]. Similarly, the radical scavenging and the anti-Aβ aggregation activity of PGG could be due to its structural characteristics, and do not necessarily indicate that its multifunctionality is linked with same mechanisms. In future studies, we hope to elucidate the detailed mechanism of action in each of these activities.

Furthermore, the neuroprotective potential of Gobaishi extract and the isolated compounds against Aβ-induced cytotoxicity was assessed in the SH-SY5Y neuroblastoma cell line. The incubation of SH-SY5Y neuroblastoma cells with Aβ induced significant cytotoxicity, consistent with previous findings that Aβ promotes neurotoxicity [46]. Also, the highly ordered aggregate pattern of Aβ42 exhibits strong membrane disruption ability which causes cell death [47]. In our study, to assess cell death we have used PI, which is a nucleic acid-binding dye which cannot penetrate the cell membrane of live cells, thus providing information about cell necrosis. Aβ monomers alone induced marked apoptosis of the cells, while co-incubation with the test compounds and extracts markedly reduced this effect. These results indicated that PGG and MG may reduce the cytotoxicity of Aβ42 through inhibiting the formation of highly ordered aggregates. Also, PGG strongly reduced cell death. This result may be attributed to its chemical structure and its anti-inflammatory and antioxidant activities. However, at high concentration (300 μM), the reducing activity was not significant. Our results align with a previous study, which reported that PGG lacks toxicity to the cells up to 50 μM, but toxicity at higher concentrations was not investigated [26].

The drug-likeness of PGG was evaluated based on Lipinski′s Rule of Five. The parameters were obtained from the SciFinder-n database, which reports values calculated using Advanced Chemistry Development (ACD/Labs) software. The retrieved data (accessed on June 21, 2025) showed that PGG has a molecular weight of 940.68 g/mol, 15 hydrogen bond donors, 26 hydrogen bond acceptors, and a logP of 5.34. These values significantly violate the Lipinski guidelines (molecular weight < 500, logP < 5, H-bond donors < 5, H-bond acceptors < 10) [48], suggesting that PGG might not fit the criteria for ideal oral drug candidates. Regarding bioavailability and metabolism, pharmacokinetic studies indicate that PGG has extremely low oral bioavailability [49]. Following oral administration in mice, plasma PGG levels were below detectable limits even at high doses (80 mg/kg), which indicates major first-pass metabolism. Less than 2% of orally administered PGG was recovered [49,50], suggesting that more than 98% was metabolized and accumulated in organs and tissues. The major excretion routes were via urine and feces. Therefore, PGG displays poor drug-likeness and exceptionally low oral bioavailability. Structural optimization or formulation strategies may be necessary to improve its potential for therapeutic applications. In contrast, MG meets all of Lipinski′s rule of five criteria (molecular weight 184.15, logP 1.24, 3 H-bond donors, 5 H-bond acceptors) [48], indicating good drug-likeness for oral application. Pharmacokinetic studies show that MG is mainly metabolized by glucuronidation and is widely distributed in tissues after administration [51,52]. Nanoformulation can further optimize its absorption and distribution.

Plant extracts consist of complex compositions of secondary metabolites that can exhibit synergistic or additive effects, significantly contributing to overall bioactivity [53]. Several studies have reported that many polyphenols, including tannins, can improve the bioactivity by their synergistic interactions [54,55]. For instance, curcumin and quercetin, together with vitamin C, have been reported to have synergistic effects on Aβ fibrillation [56]. However, our study could not investigate the mechanism of these synergistic interactions. In addition, we found that the Aβ aggregation-inhibitory activities of both isolated compounds accounted for 1.5% and 0.7% of the activity observed in the crude ethanol extract of Gobaishi, respectively, indicating that there might be other compounds present in Gobaishi, but this was not explored in this study. Factors such as the choice of solvents, temperature, and extraction duration can affect the physicochemical properties and bioactivities of plant extracts [57]. In our study, various column chromatography techniques were used for extended periods to isolate compounds, which may affect the quality of isolated compounds [58]. In our study, we could not assess the purity and extraction efficiency of the isolated compounds. Moreover, our study primarily focuses on in vitro assays, and in vivo studies are essential to confirm the therapeutic potential and safety of the extracts and isolated compounds. Lastly, this study does not explore the inhibitory mechanism of Aβ aggregation by the extracts and isolated compounds.

## 4. Materials and Methods

### 4.1. Reagents and Instruments

The organic solvents utilized in the present study, including ethyl acetate (EtOAc), methanol (MeOH), ethanol (EtOH), chloroform (CHCl_3_), acetonitrile (CH_3_CN), and dimethyl sulphoxide (DMSO), were all sourced from Kanto Chemical (Tokyo, Japan). Silica gel with a mesh size of 70–230 (Merck, Darmstadt, Germany), CHROMATOREX ODS DM1020T (Fuji Silysia Chemical, Kasugai, Japan), and Sephadex LH-20 (Merck, Darmstadt, Germany) were utilized in normal phase column chromatography, reverse-phase column chromatography, and size exclusion chromatography, respectively. For the analysis in thin-layer chromatography (TLC), silica gel 60 F254s and silica gel 60 RP18 F254s plates (Merck-Millipore, Darmstadt, Germany) were used, with UV absorption detected using the Handy UV Lamp SLUV-4 at 254 nm (AS ONE Corporation, Osaka, Japan). Anisaldehyde–sulphuric acid and 1% iron (Ⅲ) chloride solution were used as visualization reagents during TLC analysis to facilitate color development upon heating. Proton (^1^H), Carbon (^13^C), DEPT, ^1^H-^1^H COSY, HMQC, and HMBC NMR spectra were acquired using the JEOL JMN-ECA-FT-500 nuclear magnetic resonance (NMR) system. For the ^1^H NMR spectrum measurement, the methanol-*d*4 (CD_3_OD) signal was based on 3.3 ppm, while the heavy water (D_2_O) signal was based on 4.79 ppm. For the measurement of the ^13^C NMR spectrum, the (CD_3_OD) signal was referenced at 49 ppm. The coupling constant (*J*) is denoted in hertz (Hz), the chemical shift values are represented in delta (δ, ppm), and the coupling modes are annotated as follows: singlet (s), doublet (d), doublets of doublet (dd), triplet (t), multiplet (m). For liquid chromatography–mass spectrometry (LC-MS) analysis, a Shimadzu LCMS system was used for electrospray ionization mass spectrometry, with measurements conducted via direct introduction. HPLC grade CH_3_OH, CH_3_CN purchased from Kanto Chemical (Tokyo, Japan), and Milli-Q (Merck-Millipore) water were used for LC-MS analysis. Human neuroblastoma SH-SY5Y cells were obtained from KAC (Kyoto, Japan).

### 4.2. Preparation of Plant Extract

Gobaishi, which are the galls of sumac tree, were collected from the *Rhus javanica* var. *chinensis* plant and manufactured in China. These dried Gobaishi were imported and sold by Aikuma Senryo Co., Ltd. (Tokyo, Japan). This material was collected as a dye, and no special permission was required. Gobaishi were macerated into small fragments, and 386 g was soaked in 95% ethanol (*w*/*v* L × 5 times) at room temperature for one week. The resulting mixture was then filtered using Whatman No. 1 filter paper (Whatman, Maidstone, UK). The EtOH filtrate was collected and subsequently concentrated under reduced pressure by a rotary evaporator at a temperature of 35 °C, yielding 226 g (58.5%; *w*/*w*) of the EtOH extract, which was stored at 4 °C for further analysis.

### 4.3. Metabolite Analysis

The metabolite analysis of Gobaishi EtOH extract was performed using a LQT XL liner ion trap mass spectrometer (Thermo Fisher Scientific, San Jose, CA, USA) in conjunction with Vanquish Duo UHPLC (Thermo Fisher Scientific, San Jose, CA, USA) system. The mass spectrometric detection was carried out in full scan mode, covering positive (sid = 20) and negative (sid = 5; 30) ion mode, with a mass range from *m/z* 150 to 1500 amu to capture the metabolites present in the extract. The parameters for the mass analysis were as follows: capillary temp 275 °C, source heater temp 350 °C. For positive mode, the source voltage was 3.50 kV, source current 100.00 µA, capillary voltage −9.00 V. For negative mode, the source voltage was 4 kV, source current was 100.00 µA, and capillary voltage was 10 V.

The components were separated using a C18 100Å, 3 µm, 2.1 × 100 mm column. The chromatographic run started with two mobile phases consisting of 0.1% formic acid water (A) and 0.1% formic acid acetonitrile (B). The following gradient was applied at a flow rate of 200 µL/min: 0–1 min, 100% A; 1–2 min, from 100% to 90% A; 2–16 min, from 90% to 60% A; 16–22 min, from 60% to 0% A; 22–25 min, to 0% A, column wash; 25–30 min, to 100% A for equilibration of the column. The sample injection volume was 2 μL. The method showed good linearity across a concentration range of 0.001 to 10 mg/mL, with a correlation coefficient (R^2^) ≥ 0.958. For the metabolite profiling study, Xcalibur 4.1 (Thermo Fisher Scientific, San Jose, USA) was used to process the data.

### 4.4. Bioassay-Guided Fractionation

The anti-amyloid β aggregation activity of the Gobaishi EtOH extract was assessed utilizing the ThT assay. Following the evaluation of its inhibitory efficacy, a portion of the EtOH extract (30 g) was subjected to liquid–liquid partitioning employing chloroform (CHCl_3_), ethyl acetate (EtOAc), water-saturated butanol (BuOH), and water (H_2_O). The resulting fractions were then concentrated using a rotary evaporator, yielding 1.98 g of the CHCl_3_ extract, 22.42 g of the EtOAc extract, 10.58 g of the BuOH extract, and 1.44 g of the H_2_O extract, respectively.

Then, a portion of the bioactive EtOAc extract (500 mg) was subjected to column chromatography on silica gel with a mesh size of 70–230. A measure of 50 gm of silica gel was used for this chromatographic separation on an open column with dimensions 1.6 cm × 70 cm (inner diameter × length). The elution process was performed using a solvent system of CHCl_3_/MeOH (containing 0.1% formic acid) in a gradient from 90:10 to 0:100 (*v*/*v*) and finally eluted with MeOH (0.1% formic acid), resulting in the separation of five major fractions (fractions 1–5).

Notably, fraction 4 exhibited a significant reduction in amyloid β aggregation. Further purification was carried out on Sephadex LH-20 (in MeOH) by using 100 mg of group 4 fraction on a column with dimensions 2.5 cm × 92 cm (inner diameter × length) with a flow rate of 0.5 mL/min, resulting in the separation of two major fractions (fractions 4a and 4b). Then 85 mg of 4b fraction was further fractionated by open column chromatography on silica gel with a gradient of CHCl_3_/MeOH (containing 1% formic acid) ranging from 99:01 to 0:100, (*v*/*v*). The total volume of the column was approximately 50 mL, packed with 13 g of silica gel and fractionated into four groups (4b1–4b4). Further separation of 35 mg of group 4b4 was followed by repetitive preparative ODS thin-layer chromatography using 40% methanol in water with 1% formic acid and 30% acetonitrile in water, which yielded 18 mg of compound **1**. Additionally, preparative silica gel thin-layer chromatography with CHCl_3_/MeOH (1% formic acid) at a ratio of 80:20 resulted in 8.38 mg of compound **2** from 16.75 mg of group 4b2. At this point, both compounds were visible as a single peak in LC, detected under UV. Later, both compounds were subjected to the ThT assay to evaluate their inhibitory activity against Aβ aggregation.

The activity of each fraction is expressed as a percentage of the activity of EtOH extract of Gobaishi based on their EC_50_ value individually, and the contribution percentage of each fraction is determined using the following Equation (1):

(1) $$\%Equivalent=\frac{Activity\;of\;EtOH\;extract\;(mg/mL)}{Activity\;of\;each\;fraction\;(mg/mL)}\times 100\times Yield\;of\;fraction$$

### 4.5. NMR Spectroscopy

Compound **1**: A yellowish solid powder. ^1^H-NMR (500 MHz, CD_3_OD) δ 7.11 (s, 2H), 7.05 (s, 2H), 6.98 (s, 2H), 6.95 (s, 2H), 6.90 (s, 2H), 6.24 (d, *J* = 8.6 Hz, 1H), 5.91 (t, *J* = 9.7 Hz, 1H), 5.58 (m, 2H), 4.51 (d, *J* = 10.3 Hz, 1H), 4.43–4.36 (m, 2H). ^13^C-NMR (125 MHz, CD_3_OD) δ 167.9, 167.3, 166.9, 166.9, 166.2, 146.5, 146.5, 146.4, 146.4, 146.3, 140.3, 140.3, 140.1, 140, 139.9, 121.0, 120.3, 120.2, 120.2, 119.7, 110.6, 110.4, 110.4, 110.4, 110.3, 93.8, 74.4, 74.1, 72.2, 69.8, 63.1.

Compound **2**: A white to pale yellow solid substance. ^1^H-NMR (500 MHz, D_2_O) δ 6.99 (s, 2H), 3.74 (s, 3H). ^13^C-NMR (125 MHz, D_2_O) δ 169.0, 144.6, 138.1, 120.8, 109.9, 52.5.

### 4.6. LC-MS/MS Analysis

The LC-MS/MS experiments were carried out using a Shimadzu LCMS-8045 spectrometer. Both compounds **1** and **2** were prepared by dissolving them in MeOH at a concentration of 10 µg/mL. A Shim-pack Velox C18 column (2.7 µm, 2.1 mm × 150 mm; Shimadzu, Japan) was used as the stationary phase. The mobile phase was delivered at a flow rate of 0.2 mL/min, and the injection volume was 10 µL. Detection of both compounds was performed at a wavelength of 254 nm. The column and sample temperatures were 40 and 20 °C, respectively. The parameters for the mass analysis were as follows: nebulizer gas flow rate, 3 L/min; heating gas flow rate, 10 L/min; interface temperature, 300 °C; desolvation temperature, 526 °C; desolvation line (DL) temperature, 250 °C; and heat block temperature, 400 °C. The drying gas flow rate was 10 L/min. The mass resolution was set to mass-resolving quadruple Q3.

For the analysis of compound **1**, the mobile phase was employed, composed of solvent A (70% MeOH in MQ-H_2_O) and solvent B (60% MeOH in MQ-H_2_O), with a gradient elution protocol as follows: 0.1 min at 100% B; 50 min at 0% B; and stopping at 60 min.

To analyze compound **2**, a different mobile phase was employed, consisting of solvent A (1% formic acid in acetonitrile) and solvent B (100% MQ-H_2_O) with distinct gradient elution (0–5 min = 95% B, 5–10 min = 90% B, 10–15 min = 80% B, 15–20 min = 50% B, 20–25 min = 0% B) followed by ending of the flow at 35 min.

### 4.7. Bioactivity Assays

#### 4.7.1. Thioflavin T (ThT) Assay

To quantitatively assess Aβ aggregation, a detection methodology based on ThT assay was carried out as described in prior studies [7]. Briefly, Aβ42 was prepared by diluting 1 mM stock solution of Aβ42, stored in DMSO, to a concentration of 60 μM using 1 × PBS. The test compounds were serially diluted to achieve 600, 60, 6, 0.6, 0.06, and 0 μM concentrations in the assay buffer of 10% EtOH and 1 × PBS. To each well, 5 μL of diluted test samples at each concentration were combined with 5 μL 60 μM Aβ42, with each condition tested in triplicate. This procedure resulted in a final reaction volume of 10 μL containing 30 μM Aβ42 in 1 × PBS, 5% EtOH, and 3% DMSO, corresponding to final test compound concentrations of 300, 30, 3, 0.3, 0.03, and 0 μM. To facilitate the release of the Aβ complex adhered to the tube walls, a centrifugation step (flash spin) was performed using a Prism Mini centrifuge (Labnet, Edison, NJ, USA), ensuring that the Aβ solution was translocated to the bottom of the tube and mixed thoroughly. The samples were then incubated at 37 °C for 24 h to promote aggregation. Subsequently, the fluorescence intensity was quantified by adding 190 µL of the ThT solution (5 μM ThT in 50 mM glycine-NaOH buffer, pH 8.5) to each 10 μL of sample solution. Rosmarinic acid was used as a reference compound and exhibited sensitive detection of amyloid fibril inhibition, with a linear response (R^2^ ≥ 0.994) across (300–0.03) μM. All measurements were conducted in triplicate to ensure consistent repeatability and accuracy, confirming the method′s reliability.

A black microfluorescent cell (FM20B-B-25; GL Science, Tokyo, Japan) was used to measure the fluorescence intensity with a Hitachi G-4500 fluorescence spectrophotometer (Tokyo, Japan). The excitation and emission wavelength were set to 455 nm and 490 nm, respectively, to optimize the sensitivity of aggregation detection.

To evaluate potential interference from the test compounds with the ThT fluorescence signal (e.g., intrinsic fluorescence or quenching effects), control experiments were performed. Specifically, the fluorescence intensity was measured for solutions containing each compound at its respective concentration with ThT, but in the absence of amyloid fibrils. These controls confirmed that the compounds used in this study did not significantly affect the ThT fluorescence signal (Supplementary Figure S21).

EC_50_ values were determined from the inhibition curves by plotting the inhibition rate against the sample concentration using a global fit to a five-parameter logistic model of the asymmetric sigmoid and utilizing GraphPad Prism 8.4.3 (GraphPad Software, San Diego, CA, USA).

#### 4.7.2. Turbidity Assay

A turbidy assay was carried out to quantify the Aβ42 aggregation in the presence of PGG. Aβ42 (1 mM in DMSO) was diluted to 60 µM in 1 × PBS containing 10% DMSO. PGG was prepared at concentrations of 600, 60, 0.06, and 0 μM in the same buffer. In a 96-well plate, 50 μL of each PGG dilution was mixed with 50 μL of 60 μM Aβ42, resulting in a final reaction volume of 100 μL containing 30 μM Aβ42 and 300, 30, 0.03, and 0 μM of PGG concentrations per well. All conditions were tested in triplicate. Then, the samples were incubated at 37 °C for 24 h to allow aggregation. After incubation, the absorbance at 450 nm was measured using a microplate reader SH 9000 (Corona Electric) equipped with absorbance detection (200–1000 nm range). Background absorbance (buffer + DMSO, without Aβ42 and PGG) was subtracted and the aggregation of Aβ42 in the presence of PGG was quantified by measuring turbidity (ΔAbs) using the following Equation (2):

ΔAbs = Abs of (PGG + Aβ) − Abs of PGG(2)

Decreased turbidity relative to the Aβ42-only control indicates inhibition of aggregation.

#### 4.7.3. DPPH Assay

The efficacy of isolated compounds from Gobaishi in stabilizing DPPH free radicals was thoroughly assessed by adapting the methodology described in the research article [59]. In summary, the isolated compounds were dissolved in MeOH at concentrations of 125, 62.5, 31.25, 15.625, and 7.8125 µg/mL. Each sample (400 µL) was combined with 400 µL of 0.5 mM DPPH in MeOH. Subsequently, 200 µL aliquots were transferred to a 96-well microplate. Following a 30 min incubation in the dark, the absorbance was quantified at a wavelength of 517 nm. The percentage of Radical Scavenging Activity (RSA%) was calculated using the following Equation (2):

(3) $$\%RSA=\frac{Abs\;of\;control\;-\;(Abs\;of\;sample\;-\;Abs\;blank)}{Abs\;of\;control}\times 100$$

α-tocopherol, which was used as a positive control, demonstrated high sensitivity in detecting antioxidant activity at low concentrations. It showed linearity over a range of (125–7.8125) μg/mL, with R^2^ ≥ 0.994. All measurements were conducted in triplicate to ensure accuracy and reproducibility. The absorbance readings included Abs control, representing the absorbance of DPPH radical in MeOH; Abs sample, denoting the absorbance of the test sample in DPPH radical; and Abs blank, which is the absorbance of the test sample in MeOH. EC_50_ values were obtained by fitting inhibition curves to a five-parameter logistic model using GraphPad Prism 8.4.3 (GraphPad Software, San Diego, CA, USA).

#### 4.7.4. Apoptosis Assay

Human neuroblastoma SH-SY5Y cells were seeded in fibronectin-coated 96-well plates at a density of 1.2 × 10^5^ cells/well. Then, the cells were incubated overnight at 37 °C, 5% CO_2_. Test compounds and RA were prepared in DMSO to final working concentrations of 300, 30, 3, 0.3, and 0.03 μM. The final concentration of extract was about 0.5, 0.05, 0.005, 0.0005, and 0.00005 mg/mL. Cell death was induced by treating the cells with 30 μM Aβ combined with 1% propidium iodide (PI), in the presence or absence of the test compounds, RA, and extract. After 24 h of incubation at 37 °C, cell apoptosis was observed using a Ti-E inverted fluorescence microscope equipped with a DS-Ri2 color CMOS camera. Fluorescence images were analyzed using ImageJ software (ver 1.53e, NIH, Bethesda, MD, USA).

### 4.8. Statistical Analysis

Student′s *t*-test was performed to determine statistical significance between two groups.

## 5. Conclusions

Gobaishi is a well-known traditional medicinal plant with numerous medicinal properties. Two significant anti-Alzheimer′s compounds were identified from Gobaishi using a bioassay-guided isolation process: PGG and MG. Measuring the Aβ42 fibrillation inhibitory activity, the antioxidant capacity, and the cytotoxicity reduction capability of Gobaishi and the isolated compounds has demonstrated that this plant possesses potent anti-Alzheimer properties. Compounds targeting various pathways simultaneously may benefit the multifaceted nature of Alzheimer′s disease. In this regard, our future research will focus on the mechanism of Gobaishi extract and the isolated bioactive compounds in inhibiting the Aβ42 fibrillation and exploring their impact on other significant targets pertinent to managing Alzheimer′s disease (AD).

## Figures and Tables

**Table 1:** Major compounds of Gobaishi EtOH extract, identified through UHPLC–ion trap MS analyses.

Peak No.	Compounds	* RT/Min	Fragment Masses (*m*/*z*)	Molecular Weight	Molecular Formula
1	Gallic acid	5.77	168.89	170.12	C_7_H_6_O_5_
2	Digallic acid	9.79, 10.40	321.1, 169.01	322.22	C_14_H_10_O_9_
3	Galloylshikimic acid	8.05	325.04, 305.04, 168.87	326.25	C_14_H_14_0_9_
4	Monogalloyl Glucose	8.65	331.01, 168.91	332.26	C_13_H_16_O_10_
5	Digalloyl Glucose	9.16	483.09, 331.11, 285.07	484.4	C_20_H_20_O_14_
6	Trigalloyl Glucose	11.49	635.16, 483.17, 373.15, 168.92	636.5	C_27_H_24_O_18_
7	Tetragalloyl Glucose	12.67	787.23, 635.35, 393.24, 168.96	788.6	C_34_H_28_O_22_
8	Pentagalloyl Glucose	13.91	939.48, 769.49, 469.36, 169.03	940.7	C_41_H_32_O_26_
9	Hexagalloyl Glucose	14.64	1091.36, 939.48, 545.53, 469.63	1092.8	C_48_H_36_O_30_
10	Heptagalloyl Glucose	15.58	1243.48, 1091.55, 621.68, 545.59, 469.67	1244.9	C_55_H_40_O_34_
11	Octagalloyl Glucose	16.28	1395.45, 1243.32, 697.48, 621.38, 545.38, 469.52	1396	C_62_H_44_O_38_

* RT: retention time.

**Table 2:** Inhibitory activity of Gobaishi EtOH extracts and its subfractions obtained by liquid partition.

Fractions	Percentage of Yield (%)	* EC_50_ (mg/mL ± SD)
95% EtOH extract	58.5	1.65 ± 0.11
CHCl_3_ fraction	5.4	n.a.
EtOAc fraction	61.5	2.376 ± 0.93
n-Butanol fraction	29	10.89 ± 4.23
Aqueous fraction	3.9	n.a.

* EC_50_: 50% Effective Concentration. Each EC_50_ value is the mean of at least three independent determinations. n.a. stands for not active (EC_50_ ≥ 1000).

**Table 3:** ^1^H, ^13^C, and HMBC data of compound **1**.

Position	δH	δC	HMBC (H-C)	DEPT
Glucose Moiety				
1	6.24(d, *J* = 8.6 Hz, 1H)	93.8	C3, C7′ (Gal 1)	CH
2	5.64–5.57 (m, 2H)	72.2	C1, C3, C4, C5, C7′ (Gal 2)	CH
3	5.91 (t, J = 9.7 Hz, 1H)	74.1	C2, C4, C7′ (Gal 3)	CH
4	5.64–5.57 (m, 2H),	69.8	C1, C2, C3, C5, C6, C7′ (Gal 4)	CH
5	4.43–4.36 (m, 2H)	74.4	C3, C4	CH
6a/6b	4.43–4.36 (m, 2H), 4.51 (d, *J* = 10.3 Hz, 1H)	63.1	C4, C7′ (Gal 5)	CH_2_
Galloyl moiety 1				
1′		121		
2′/6′	7.05 (s, 2H)	110.6	C1′, C3′, C4′, C5′, C7′ (Gal 1)	CH
3′/5′		146.5		
4′		140		
7′		166.2		
Galloyl moiety 2				
1′		120.3		
2′/6′	6.90 (s, 2H)	110.4	C1′, C3′, C4′, C5′, C7′ (Gal 2)	CH
3′/5′		146.5		
4′		140.3		
7′		167.3		
Galloyl moiety 3				
1′		120.2		
2′/6′	6.98 (s, 2H)	110.4	C1′, C3′, C4′, C5′, C7′ (Gal 3)	CH
3′/5′		146.4		
4′		140.3		
7′		166.9		
Galloyl moiety 4				
1′		120.2		
2′/6′	6.95 (s, 2H)	110.4	C1′, C3′, C4′, C5′, C7′ (Gal 4)	CH
3′/5′		146.4		
4′		140.1		
7′		166.9		
Galloyl moiety 5				
1′		119.7		
2′/6′	7.11 (s, 2H)	110.3	C1′, C3′, C4′, C5′, C7′ (Gal 5)	CH
3′/5′		146.3		
4′		140		
7′		167.9		

**Table 4:** ^1^H, ^13^C, and HMBC data of MG.

Position	δH	δC	HMBC (H → C)	DEPT
C1		120.8		
C2/C6	6.99 (s, 2H)	109.9	C1, C3, C4, C5, C7	CH
C3/C5		144.6		
C4		138.1		
C7		169.0		
C8	3.74 (s, 3H)	52.5	C7	CH_3_

**Table 5:** DPPH radical scavenging activity of isolated compounds.

Test Sample	EC_50_ (µM ± SD) *	*p*-Value
PGG	1.16 ± 0.025	9.99 × 10^−8^
MG	6.44 ± 0.13	7.65 × 10^−6^
α-tocopherol	3.19 ± 0.013	

* Each value of EC_50_ is the mean of the three independent determinations.

## Data Availability

The data presented in this study are available in article and Supplementary Materials.

## Supplementary Materials

The following supporting information can be downloaded at: https://www.mdpi.com/article/10.3390/molecules30132720/s1, Figure S1: Negative Mass spectra of the eleven main compound; Figure S2: ^1^H NMR spectrum of compound **1** acquired in CD_3_OD (500 MHz); Figure S3: ^1^H-^1^H COSY NMR spectrum of compound **1** acquired in CD_3_OD; Figure S4: ^13^C NMR spectrum of compound **1** acquired in CD_3_OD (125 MHz), Figure S5: DEPT-135 NMR spectrum of compound **1** acquired in CD_3_OD; Figure S6: DEPT-90 NMR spectrum of compound **1** acquired in CD_3_OD, Figure S7: HMQC NMR spectrum of compound **1** acquired in CD_3_OD; Figure S8: HMBC NMR spectrum of compound **1** acquired in CD_3_OD; Figure S9: LC-ESI-MS analysis of compound **1**; Figure S10: ^1^H NMR spectrum of compound **2** acquired in D_2_O (500 MHz); Figure S11: ^1^H-^1^H COSY NMR spectrum of compound **2** acquired in D_2_O; Figure S12: ^13^C NMR spectrum of compound **2** acquired in D_2_O (125 MHz); Figure S13: DEPT-135 NMR spectrum of compound **2** acquired in D_2_O; Figure S14: DEPT-90 NMR spectrum of compound **2** acquired in D_2_O; Figure S15: HMQC NMR spectrum of compound **2** acquired in D_2_O; Figure S16: HMBC NMR spectrum of compound **2** acquired in D_2_O; Figure S17: LC-ESI-MS analysis of compound **2**; Figure S18: PI Fluorescence images obtained from the SH-SY5Y cells; Figure S19: Inhibition of Aβ42 aggregation by rosmarinic acid; Figure S20: UV detection of PGG and MG in Liquid Chromatography; Figure S21: Effect of the compounds on ThT fluorescence in presence or absence of Aβ.

## Abbreviations

The following abbreviations are used in this manuscript:

AD	Alzheimer′s Disease
Aβ	Amyloid Beta
PGG	Pentagalloyl Glucose
MG	Methyl Gallate
ThT	Thioflavin T
DPPH	2,2-Diphenyl-1-picrylhydrazyl
NMR	Nuclear Magnetic Resonance
HMQC	Heteronuclear Multiple Quantum Correlation
HMBC	Heteronuclear Multiple Bond Correlation
COSY	Homonuclear Correlation Spectroscopy
DEPT	Distortionless Enhancement by Polarization Transfer
UHPLC	Ultra-High Performance Liquid Chromatography
ESI	Electrospray Ionization
MS	Mass Spectrometry
AChE	Acetylcholinesterase
BChE	Butyrylcholinesterase
BACE1	Beta-Site APP Cleaving Enzyme 1
ROS	Reactive Oxygen Species
RSA	Radical Scavenging Activity
RA	Rosmarinic Acid
