# Supplementary material for: Anti-Amyloid Aggregation Effects of Gobaishi (Galla chinensis) and Its Active Constituents
Source: Molecules. 2025 Jun 24;30(13):2720. doi: 10.3390/molecules30132720 (PMC12250624; doi:10.3390/molecules30132720)
Supplement: Supplementary file 1 [file molecules-30-02720-s001.zip › molecules-3664091-supplementary.pdf]

Supporting Information for

Anti-Amyloid Aggregation Effects of Gobaishi (*Galla chinensis*) and Its Active Constituents

Sharmin Akter [1], Takayuki Tohge [2], Sahithya Hulimane Ananda [3], Masahiro Kuragano [3], Kiyotaka Tokuraku [3] and Koji Uwai [1,*]

[1] Laboratory of Organic Chemistry in Life Science, Muroran Institute of Technology, Muroran 050-8585, Japan; 22096504@muroran-it.ac.jp
[2] Laboratory of Plant Secondary Metabolism, Nara Institute of Science and Technology, Nara 630-0192, Japan; tohge@bs.naist.jp
[3] Laboratory of Protein Chemistry, Muroran Institute of Technology, Muroran 050-8585, Japan; 22096502@muroran-it.ac.jp (S.H.A.), gano@muroran-it.ac.jp (M.K.), tokuraku@muroran-it.ac.jp (K.T.)
* Correspondence: uwai@muroran-it.ac.jp

Figure S1. Negative Mass spectra of the eleven main compound. ………………………………………………...2

Figure S2. ^{1}H NMR spectrum of compound 1 acquired in CD_3OD (500 MHz)…………………………………..6

Figure S3. ^{1}H-^{1}H COSY NMR spectrum of compound 1 acquired in CD_3OD……………………………………6

Figure S4. ^{13}C NMR spectrum of compound 1 acquired in CD_3OD (125MHz) ……………………………….7

Figure S5. DEPT-135 NMR spectrum of compound 1 acquired in CD_3OD………………………………………7

Figure S6. DEPT-90 NMR spectrum of compound 1 acquired in CD_3OD………………………………………...8

Figure S7. HMQC NMR spectrum of compound 1 acquired in CD_3OD…………………………………………..8

Figure S8. HMBC NMR spectrum of compound 1 acquired in CD_3OD…………………………………………...9

Figure S9. LC-ESI-MS analysis of compound 1…………………………………………………………………9

Figure S10. ^{1}H NMR spectrum of compound 2 acquired in D_2O (500 MHz)……………………………………10

Figure S11. ^{1}H-^{1}H COSY NMR spectrum of compound 2 acquired in D_2O. ……………………………………10

Figure S12. ^{13}C NMR spectrum of compound 2 acquired in D_2O (125 MHz)……………………………………11

Figure S13. DEPT-135 NMR spectrum of compound 2 acquired in D_2O. ………………………………………11

Figure S14. DEPT-90 NMR spectrum of compound 2 acquired in D_2O……………………………………………12

Figure S15. HMQC NMR spectrum of compound 2 acquired in D_2O……………………………………………12

Figure S16. HMBC NMR spectrum of compound 2 acquired in D_2O……………………………………………13

Figure S17. LC-ESI-MS analysis of compound 2…………………………………………………………………13

Figure S18. PI Fluorescence images obtained from the SH-SY5Y cells….…………………………………………...14

Figure S19. Inhibition of Aβ42 aggregation by rosmarinic acid…………………………………………………..14

Figure S20. UV detection of PGG and MG in Liquid Chromatography……………………………………….....15

Figure S21. Effect of the compounds on ThT fluorescence in presence or absence of Aβ……………...............15

**1.**	**RT: 5.77 min** 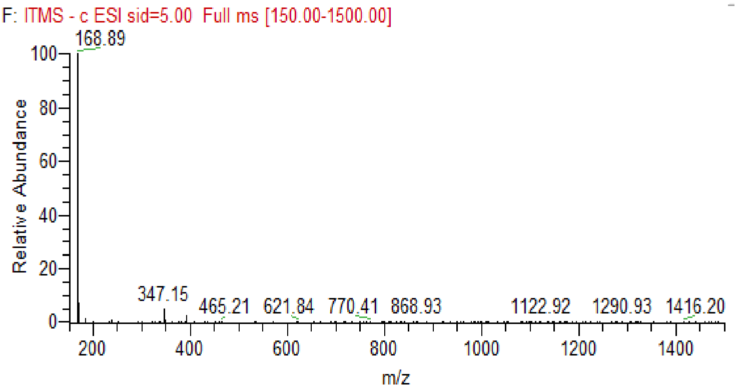	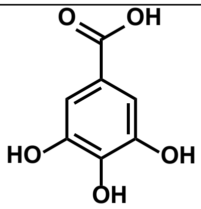 Gallic acid
**2.**	**RT: 9.79 min** 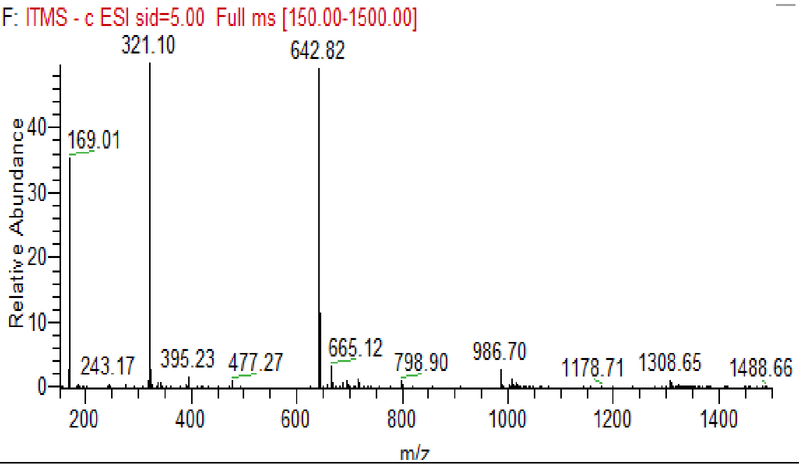	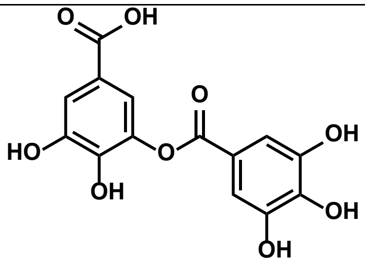 Digallic acid
**3.**	**RT: 8.05 min** 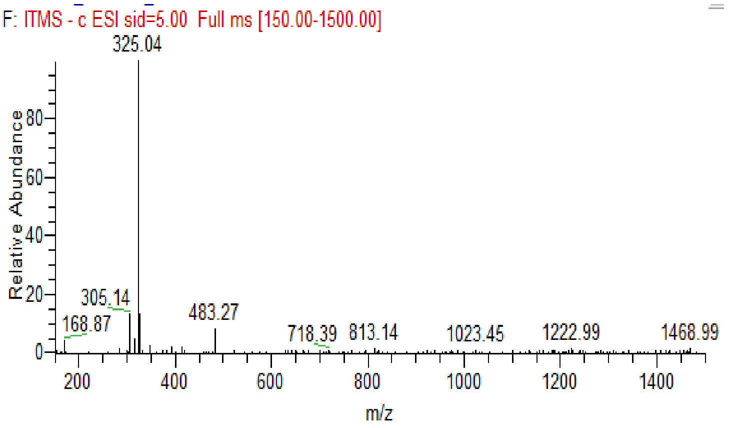	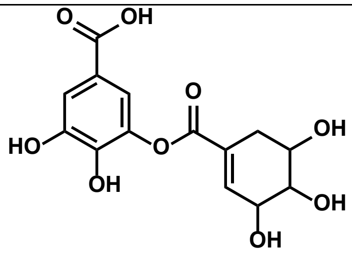 Galloylshikimic acid

**4.**	**RT: 8.65 min** 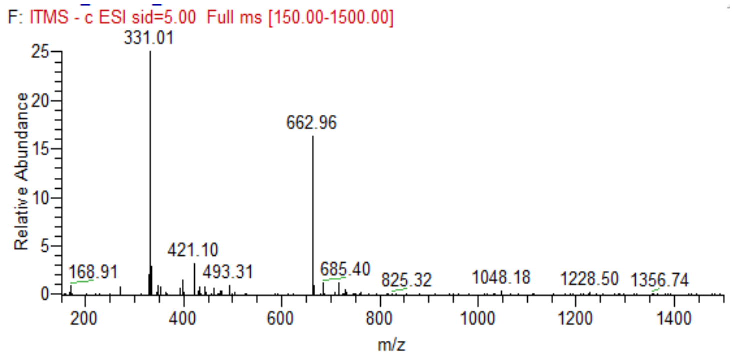	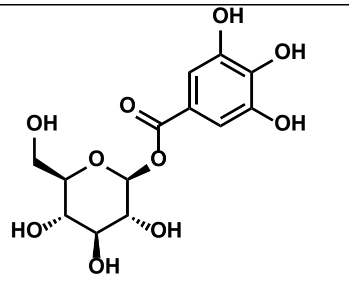 Monogalloyl glucose
**5.**	**RT: 9.16 min** 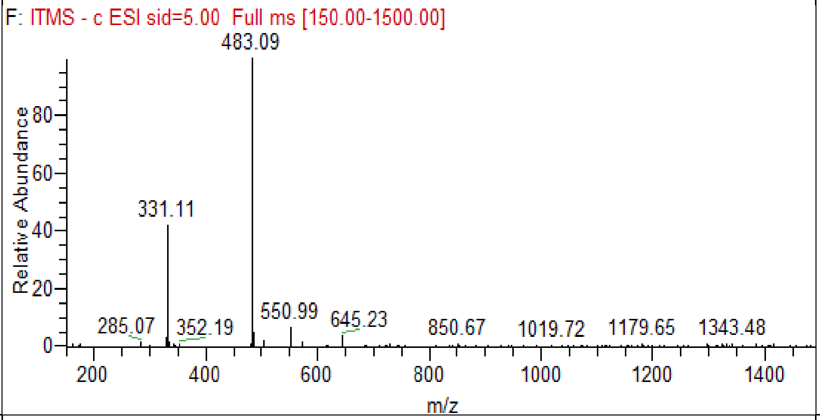	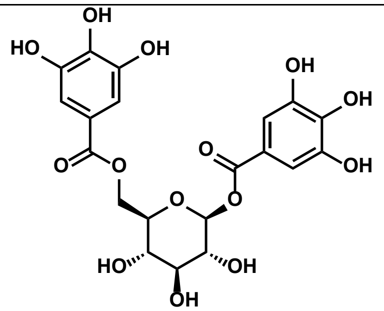 Digalloyl glucose
**6.**	**RT: 11.49 min** 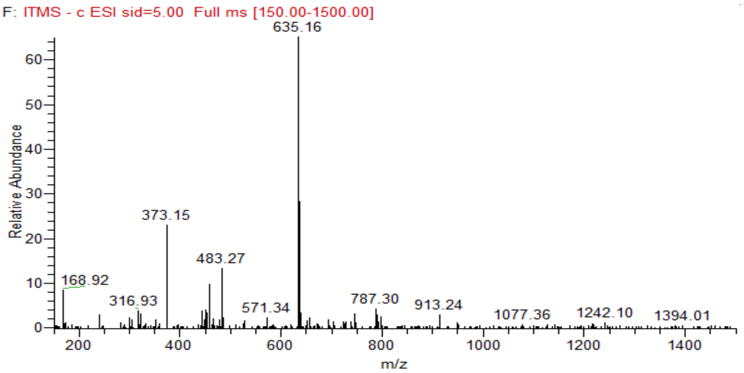	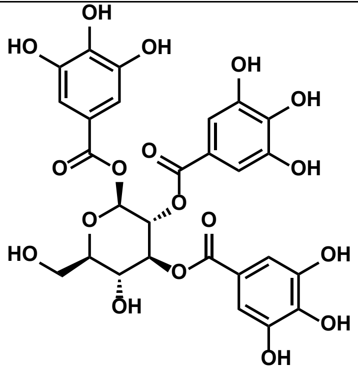 Trigalloyl glucose

**7.**	**RT: 12.67 min** 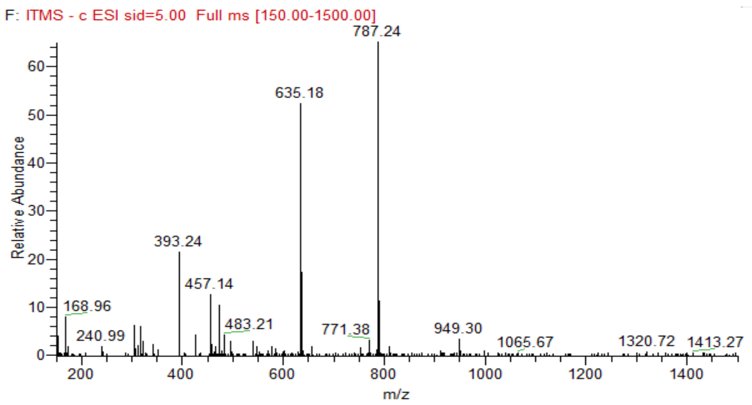	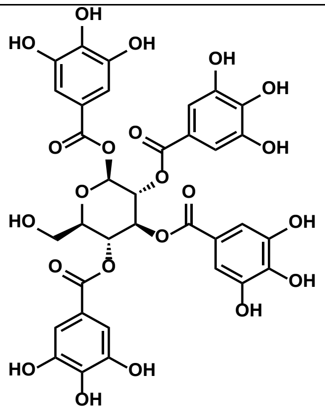 Tetragalloyl glucose
**8.**	**RT: 13.91 min** 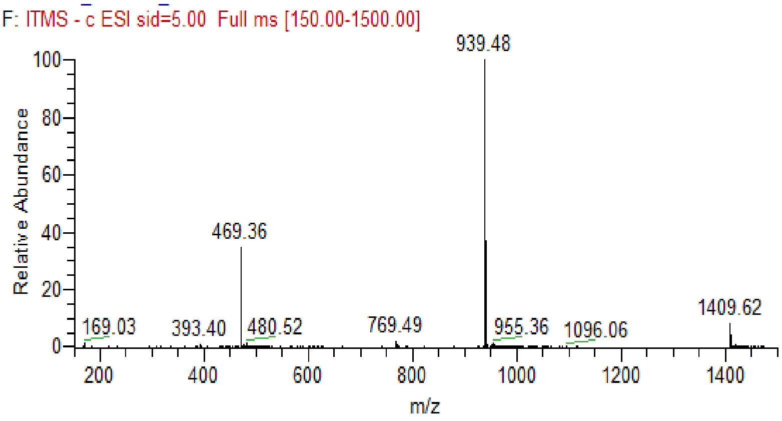	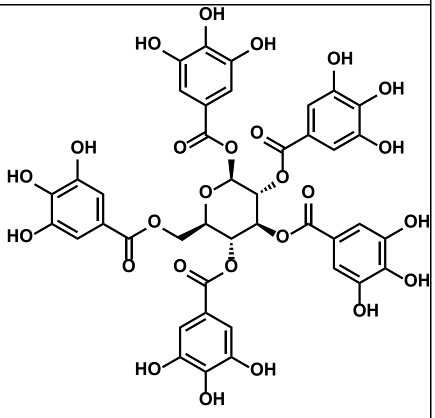 Pentagalloyl glucose
**9.**	**RT: 14.64 min** 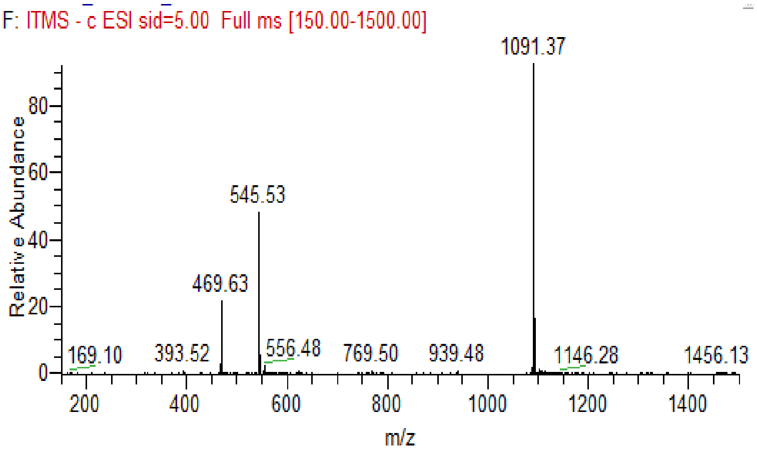	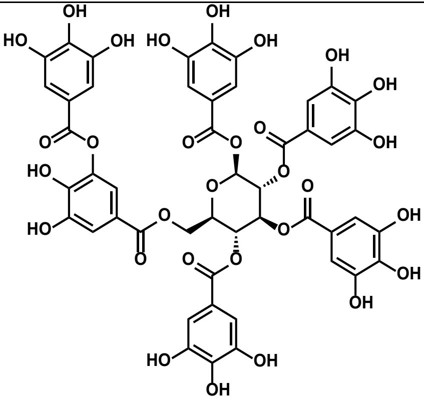 Hexagalloyl glucose

**10.**	**RT: 15.58 min**<br>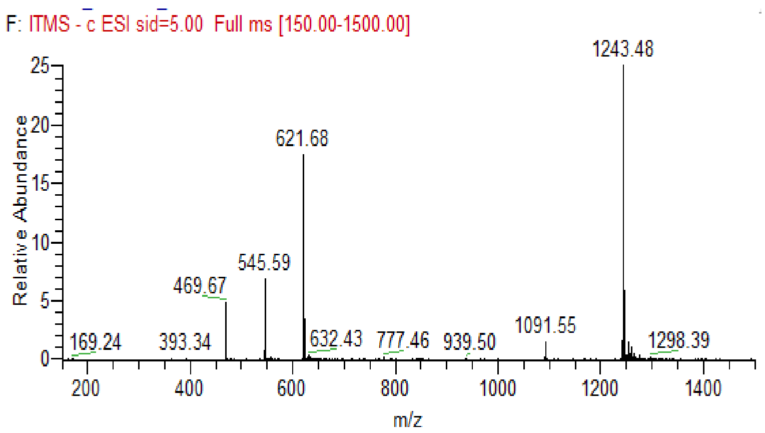 	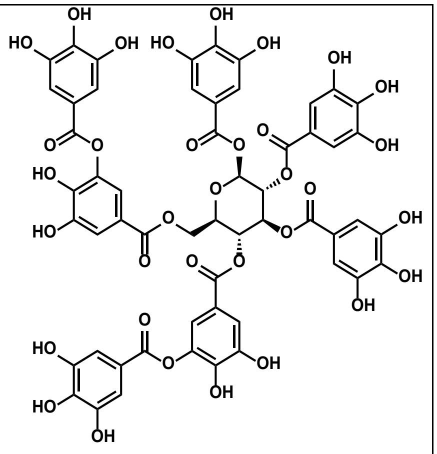<br><br>Heptagalloyl glucose
**11.**	**RT: 16.28 min**<br>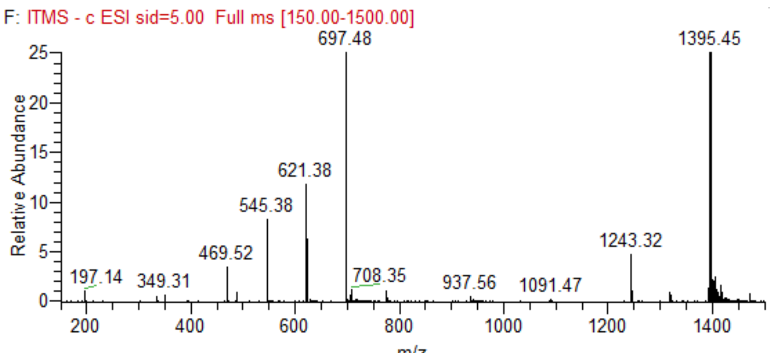 	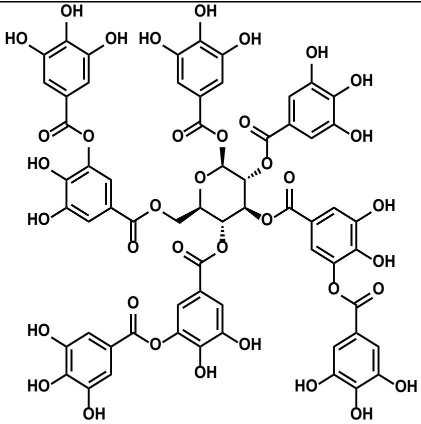<br><br>Octagalloyl glucose

Figure S1. Negative Mass spectra of the eleven main compound. (1-11), show the peaks and their corresponding molecular structure.

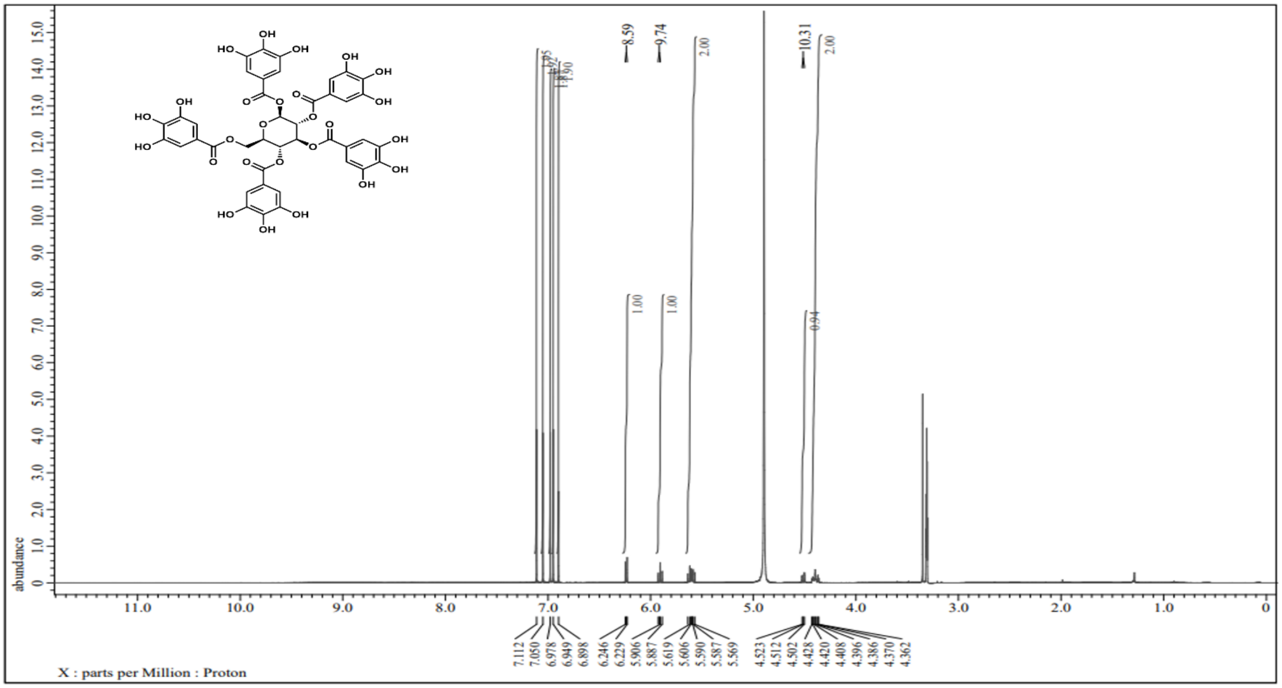

Figure S2. ^{1}H NMR spectrum of compound 1 acquired in CD_3OD (500 MHz).

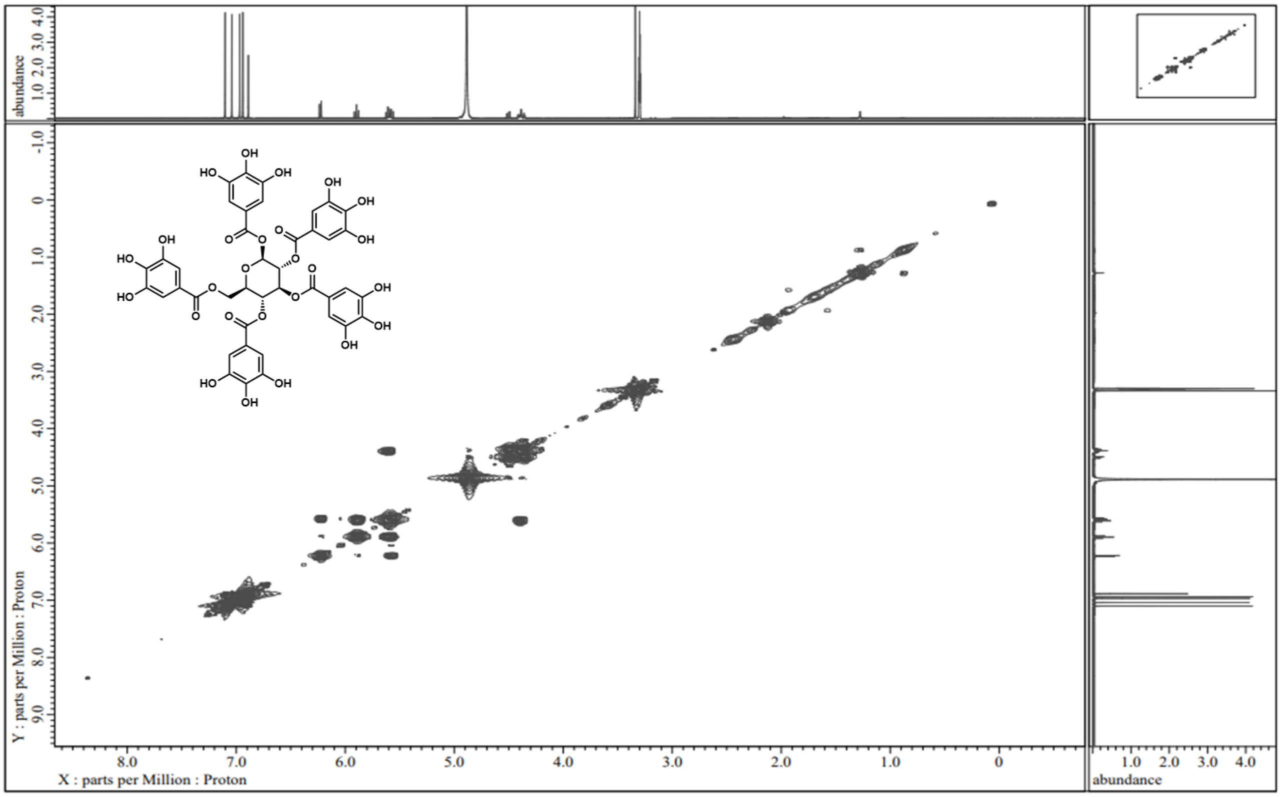

Figure S3. ^{1}H-^{1}H COSY NMR spectrum of compound 1 acquired in CD_3OD.

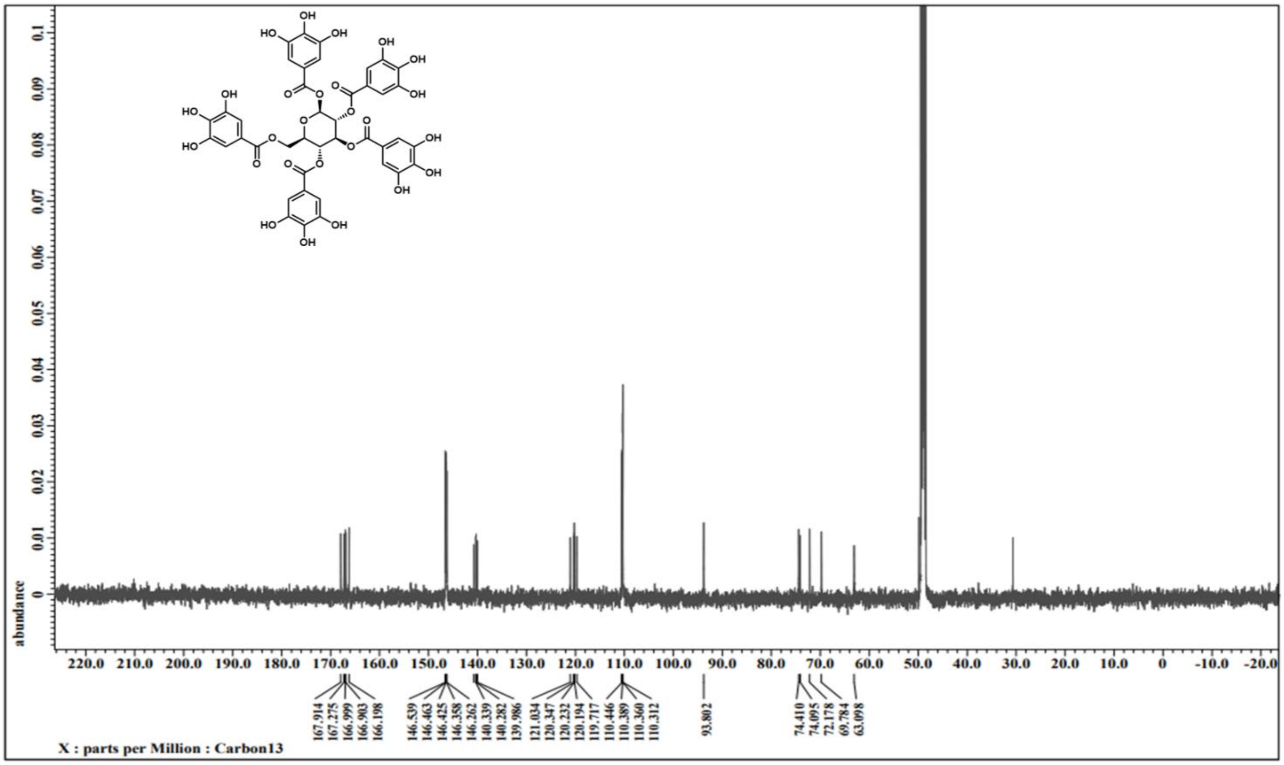

Figure S4. ^{13}C NMR spectrum of compound 1 acquired in CD_3OD (125MHz)

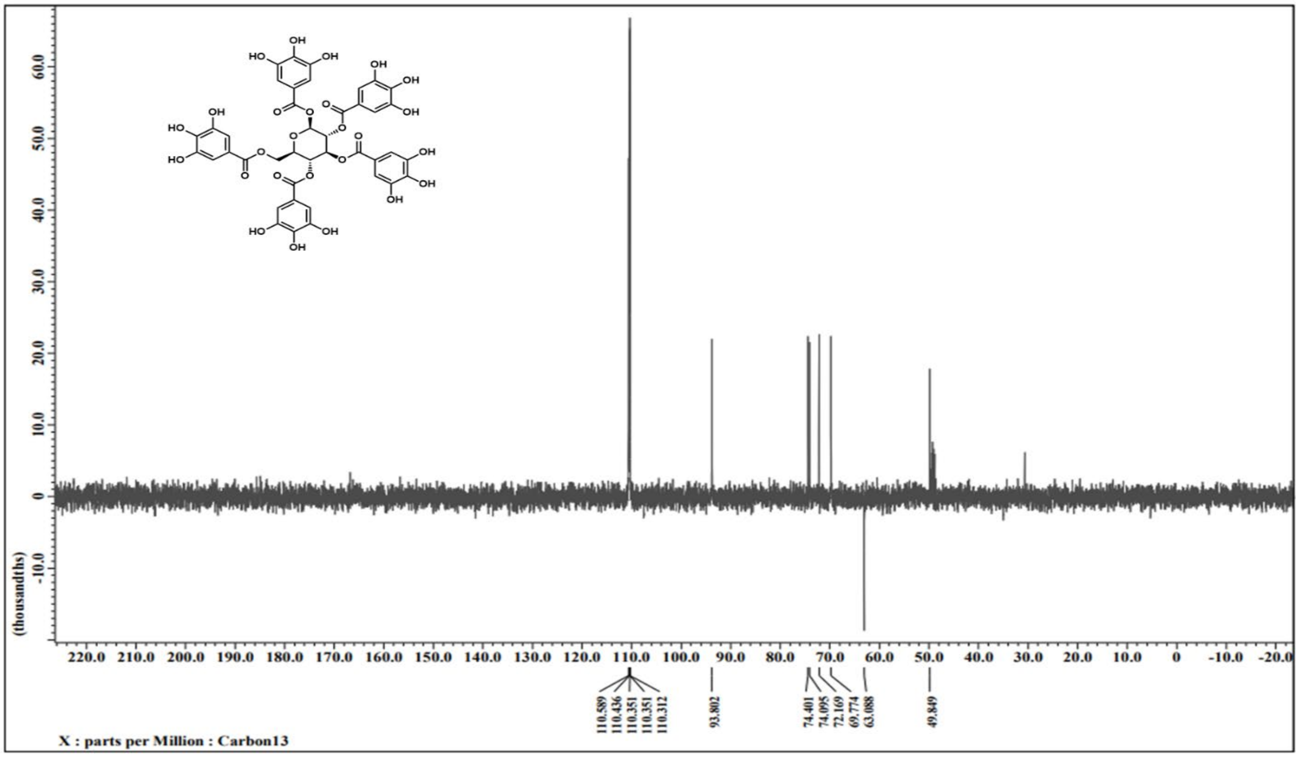

Figure S5. DEPT ^{13}C-135 NMR spectrum of compound 1 acquired in CD_3OD.

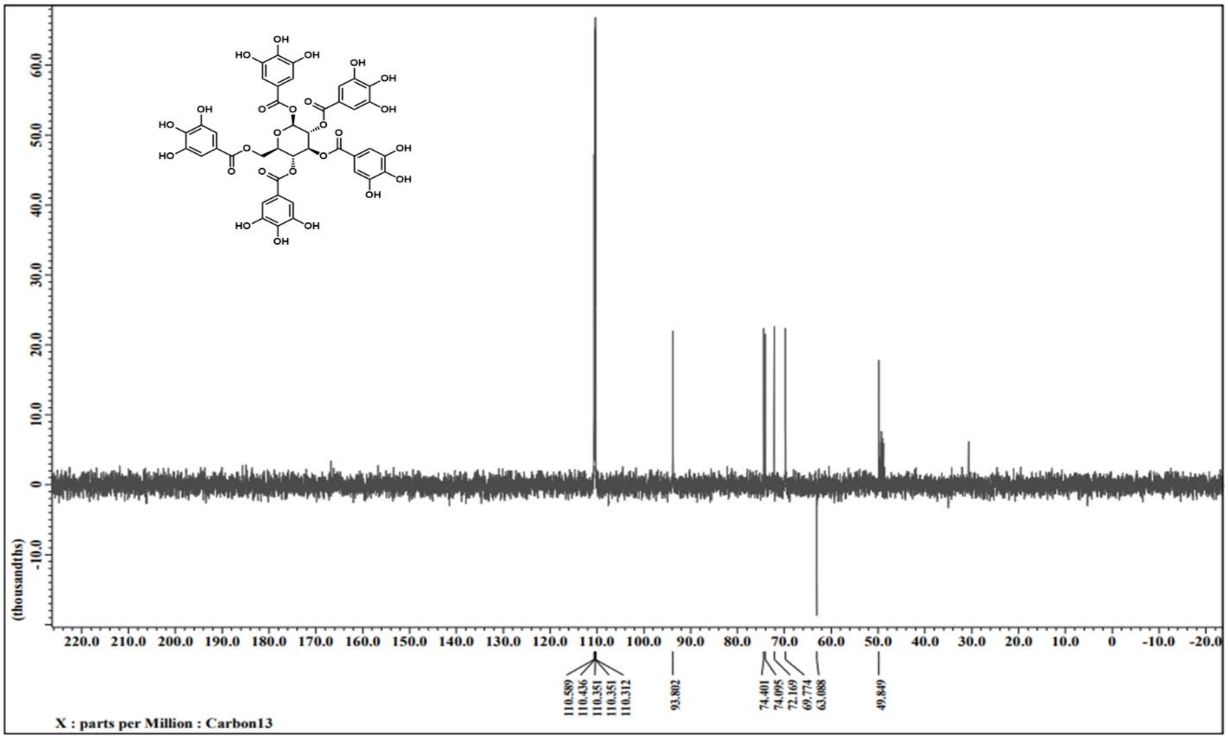

Figure S6. DEPT ^{13}C-90 NMR spectrum of compound 1 acquired in CD_3OD.

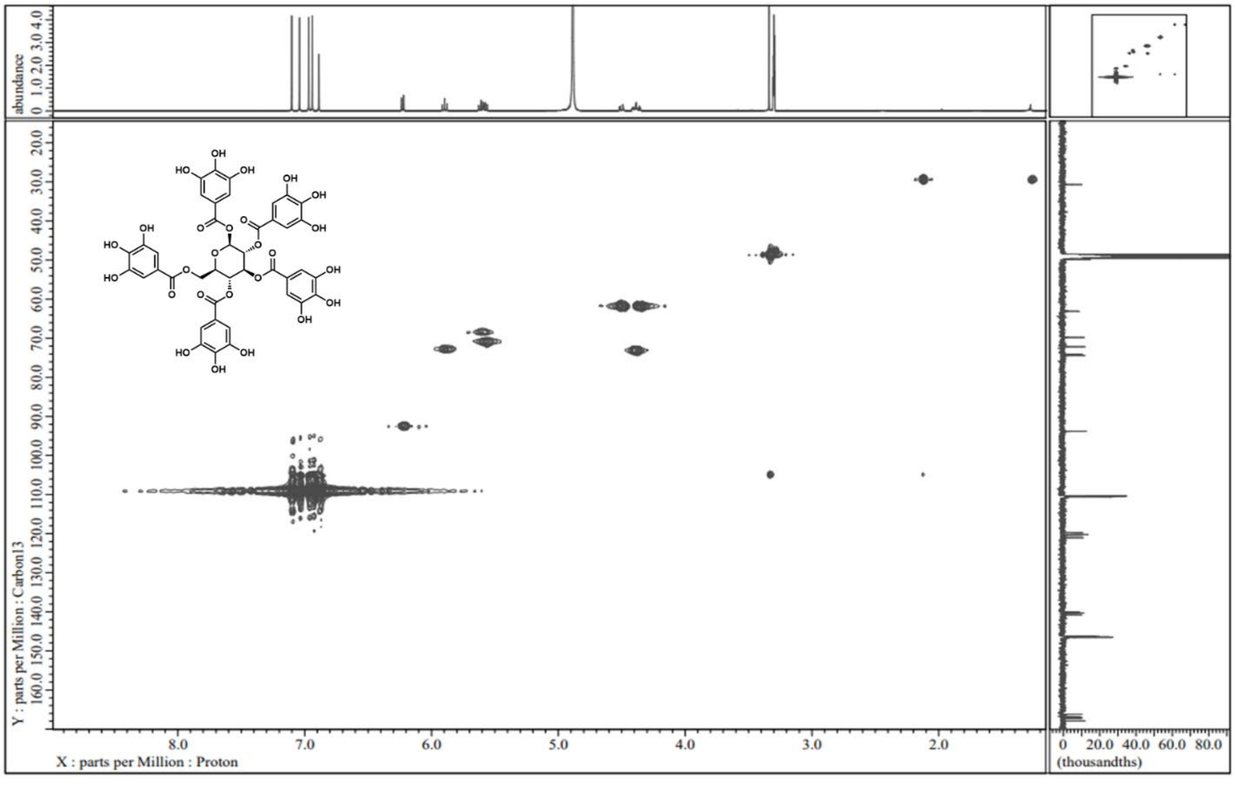

Figure S7. HMQC NMR spectrum of compound 1 acquired in CD_3OD.

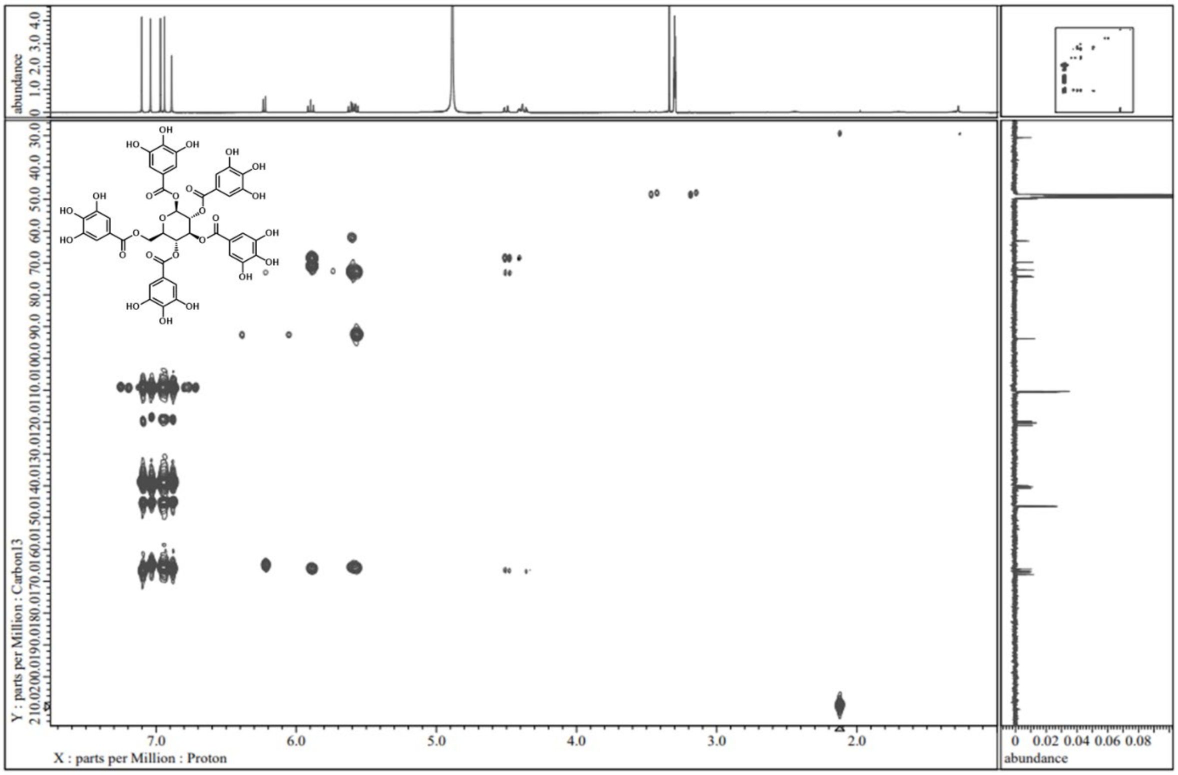

Figure S8. HMBC NMR spectrum of compound 1 acquired in CD_3OD.

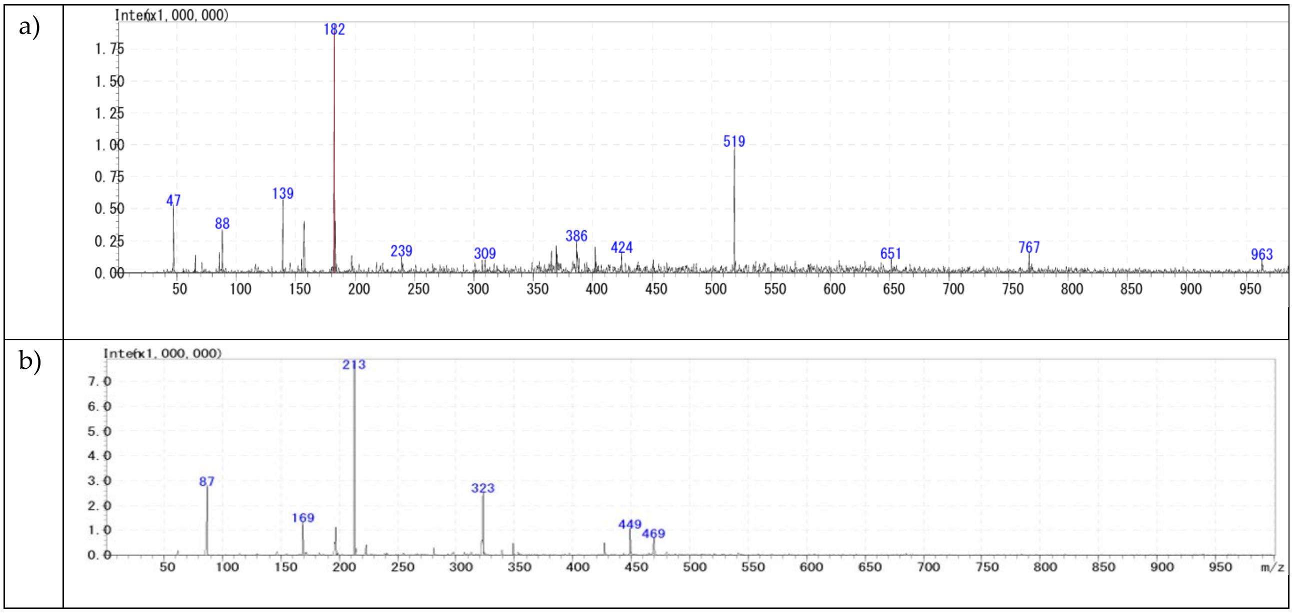

Figure S9. LC-ESI-MS analysis of compound 1; a) MS spectrum in positive mode; c) b) MS spectrum in Negative mode.

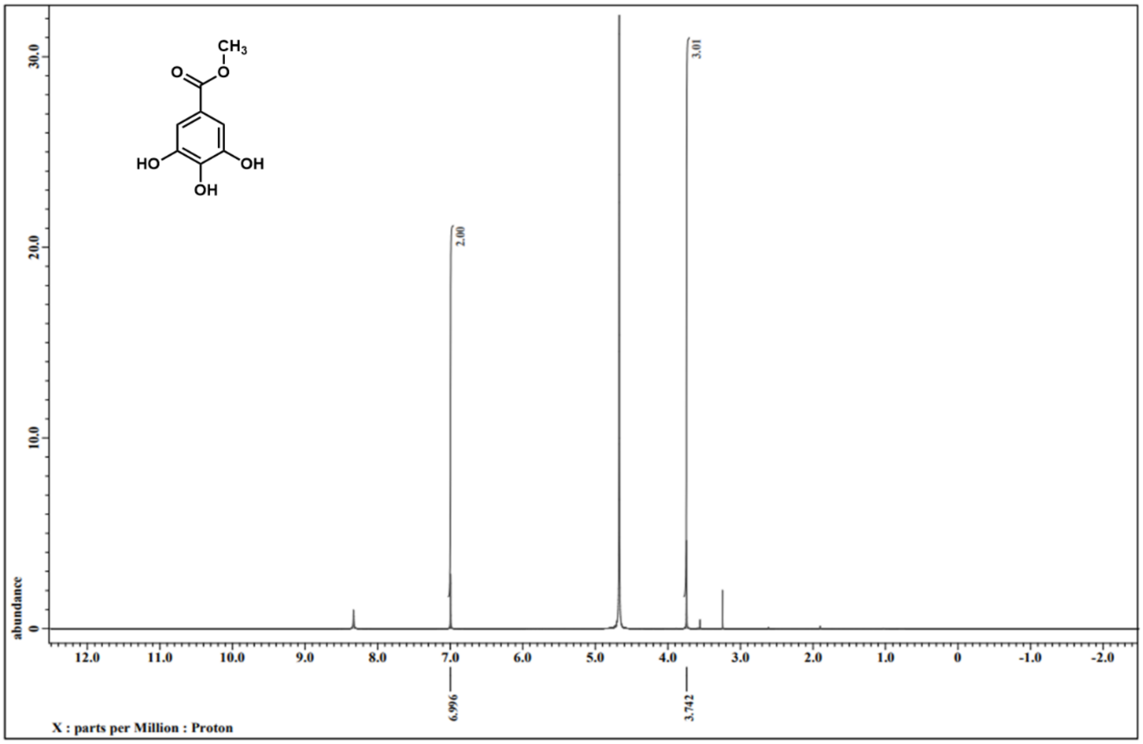

Figure S10. ^{1}H NMR spectrum of compound 2 acquired in D_2O (500 MHz).

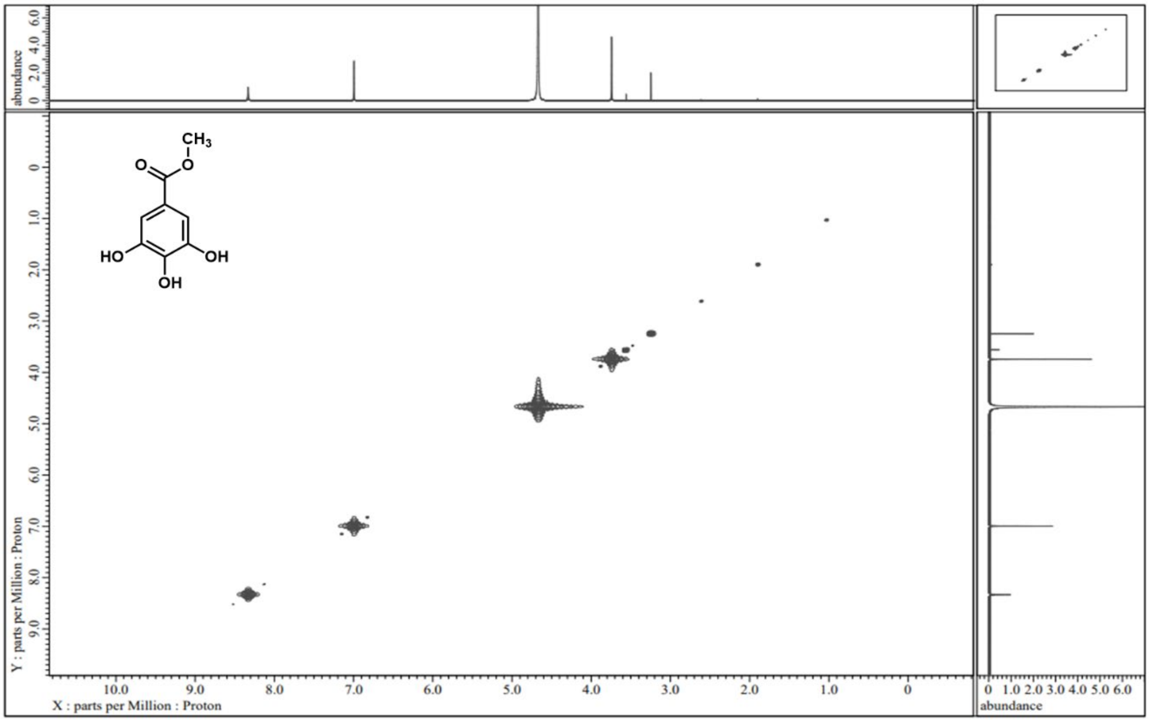

Figure S11. ^{1}H-^{1}H COSY NMR spectrum of compound 2 acquired in D_2O.

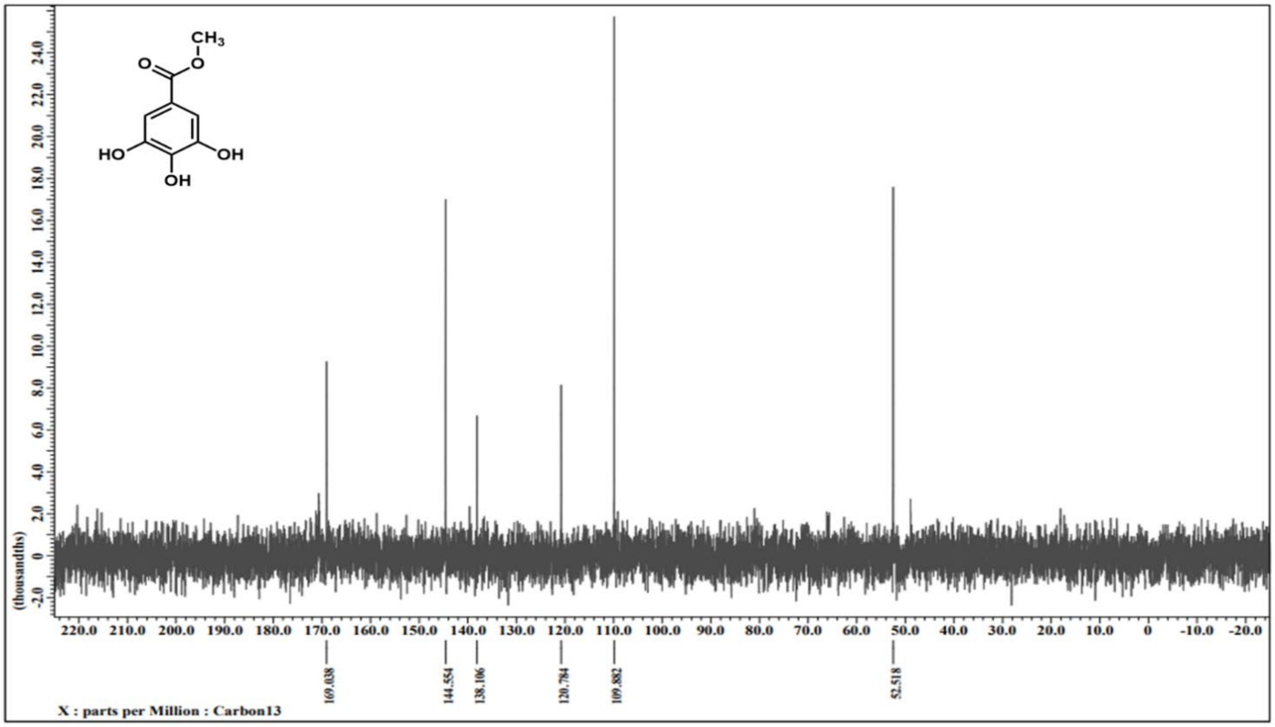

Figure S12. ^{13}C NMR spectrum of compound 2 acquired in D_2O (125 MHz).

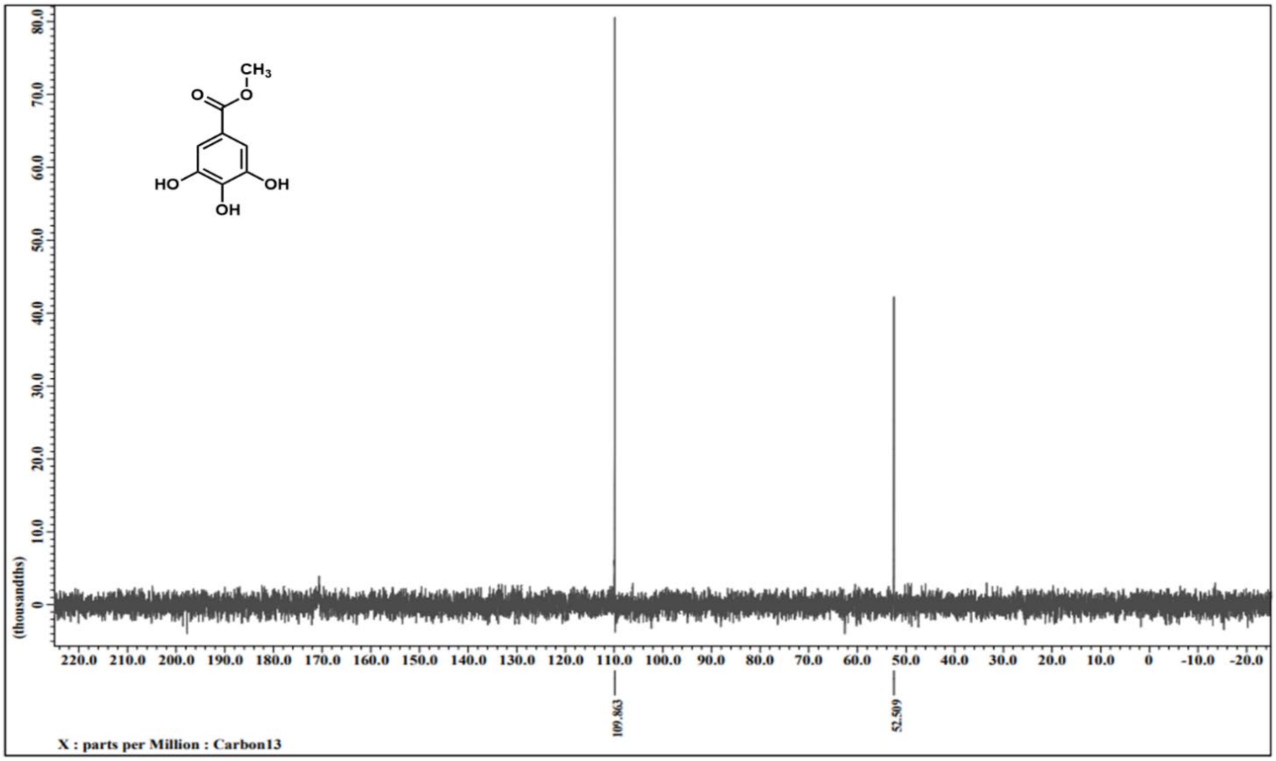

Figure S13. DEPT-135 NMR spectrum of compound 2 acquired in D_2O.

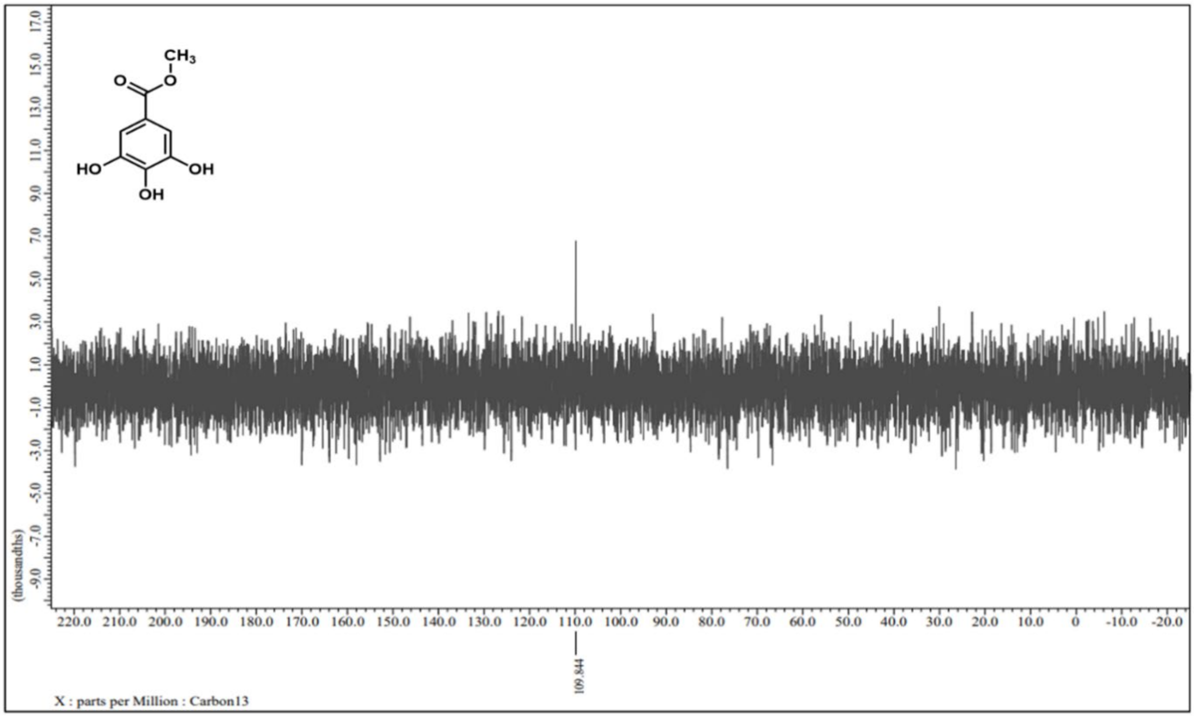

Figure S14. DEPT-90 NMR spectrum of compound 2 acquired in D_2O.

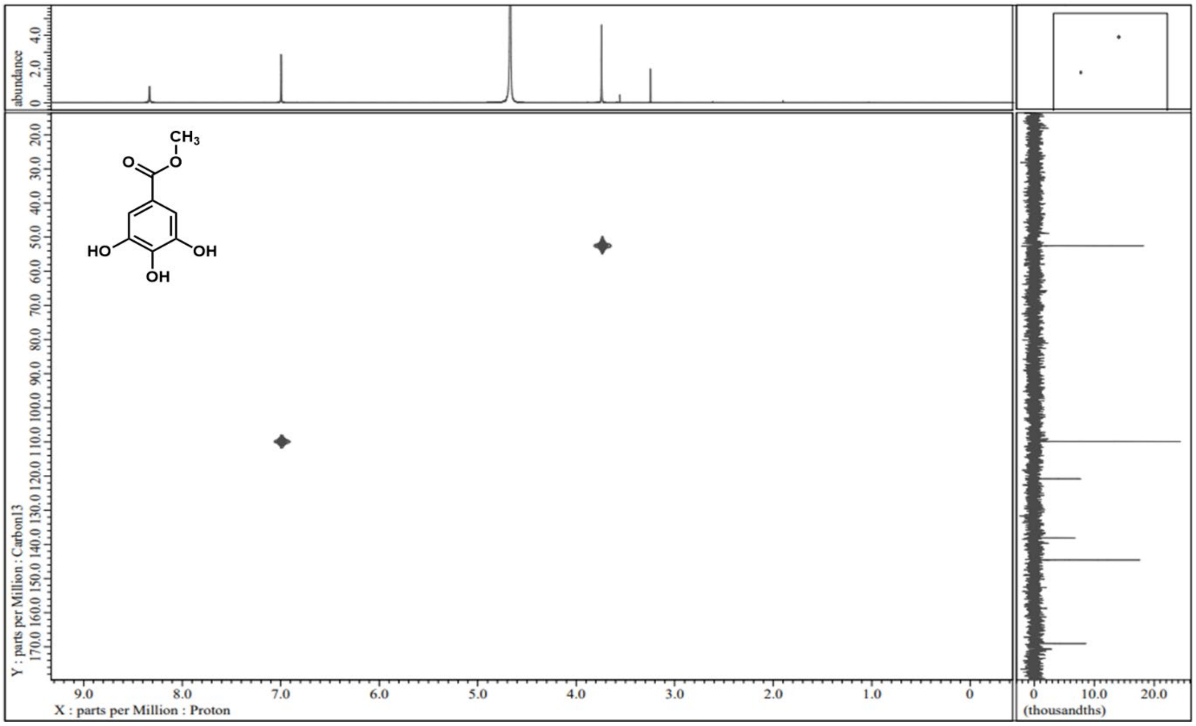

Figure S15. HMQC NMR spectrum of compound 2 acquired in D_2O.

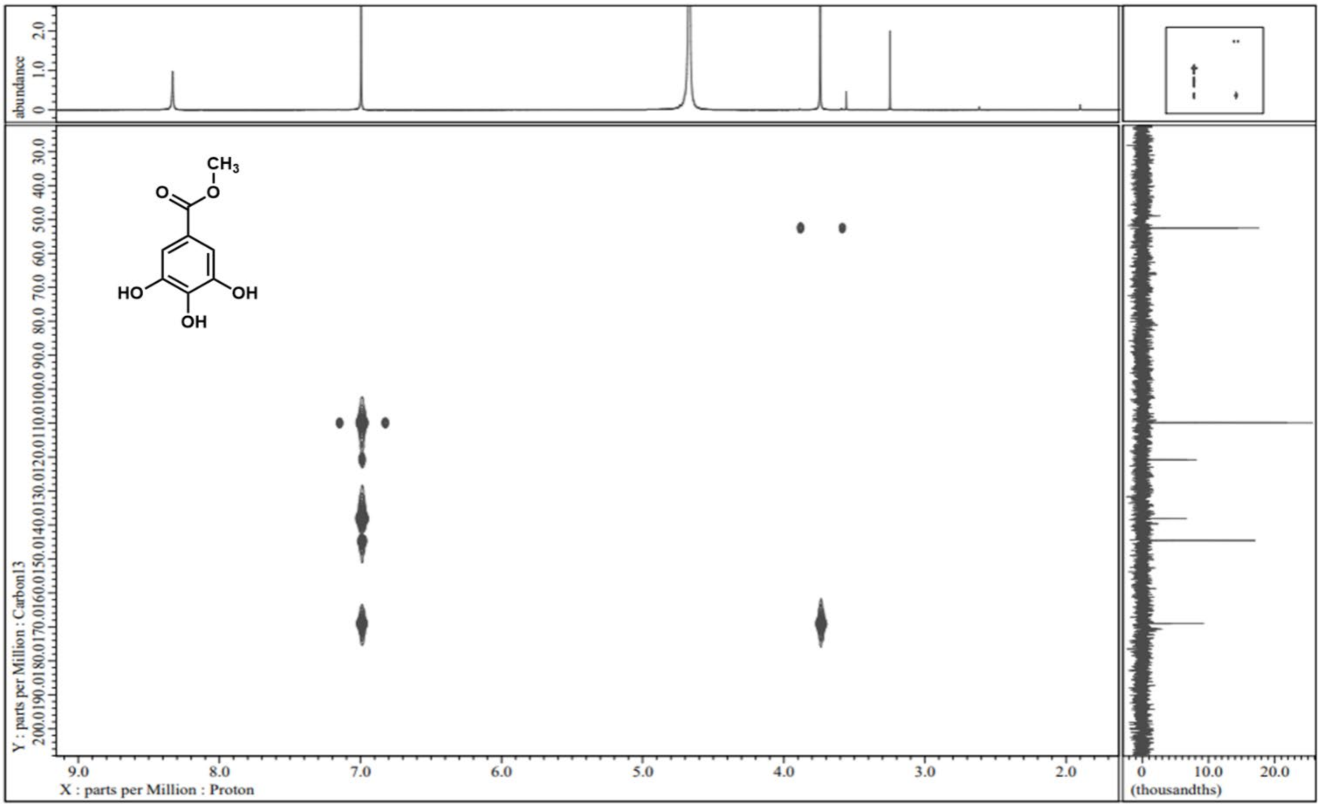

Figure S16. HMBC NMR spectrum of compound 2 acquired in D_2O.

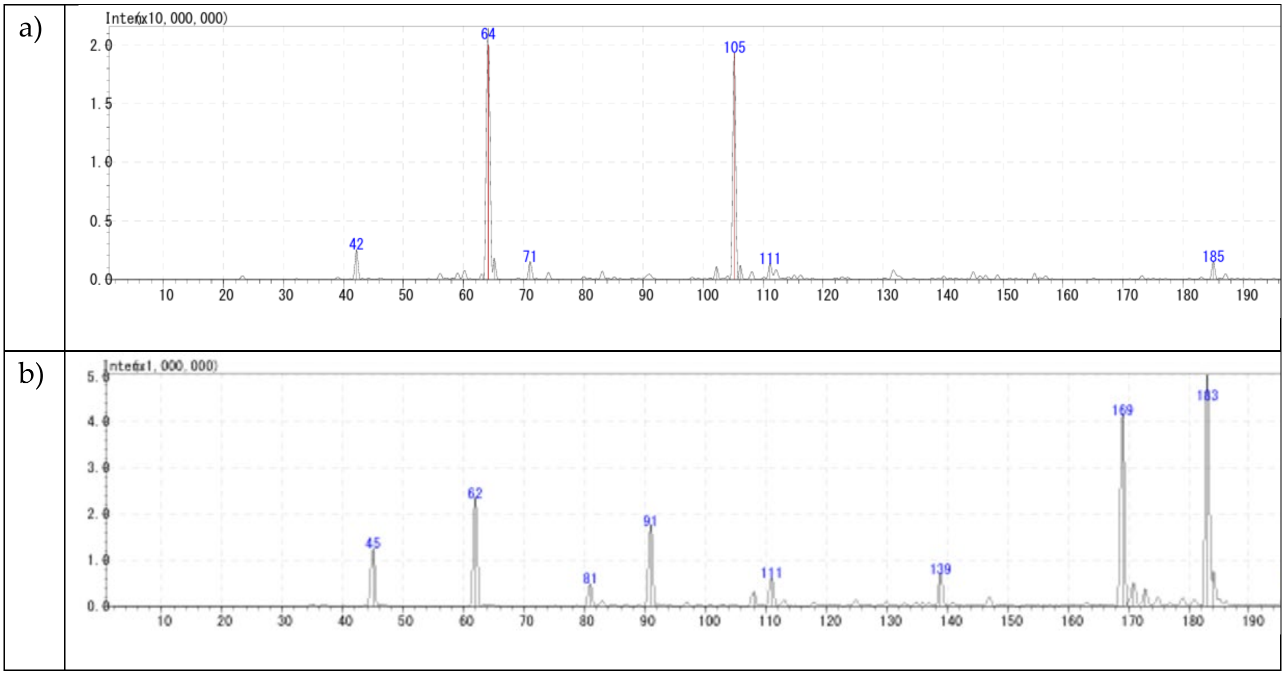

Figure S17. LC-ESI-MS analysis of compound 2; a) MS spectrum in positive mode. b) MS spectrum in negative mode.

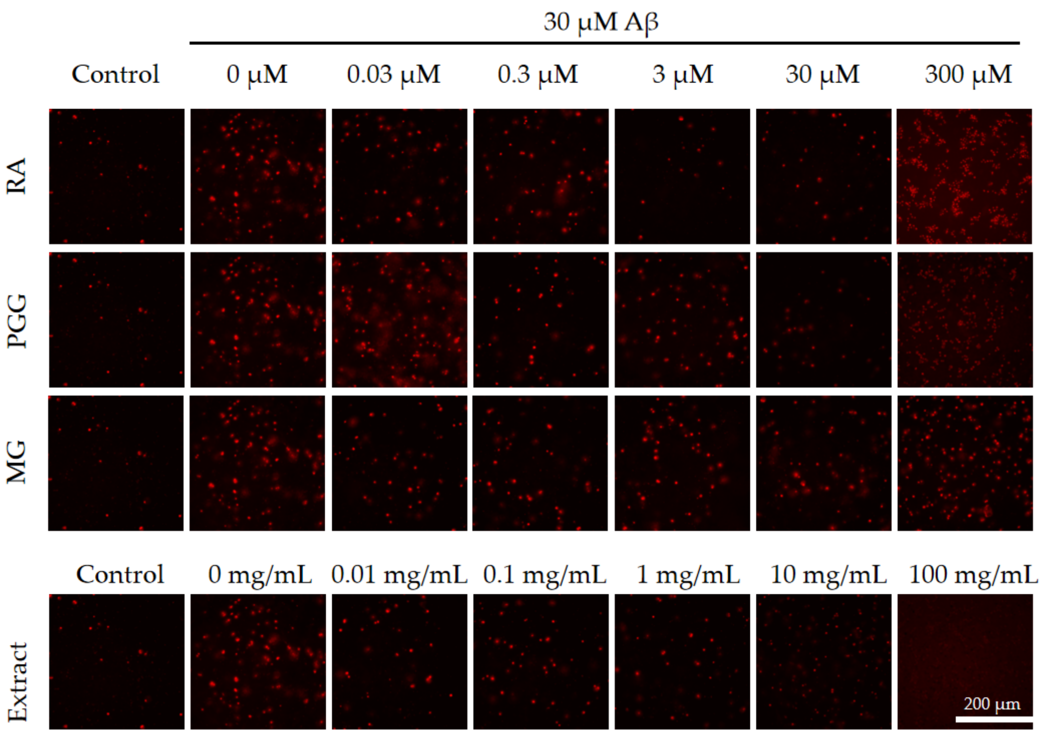

Figure S18. PI Fluorescence images obtained from the SH-SY5Y cells, co-cultured with 30 μM Aβ and different concentrations of test compounds and extract.

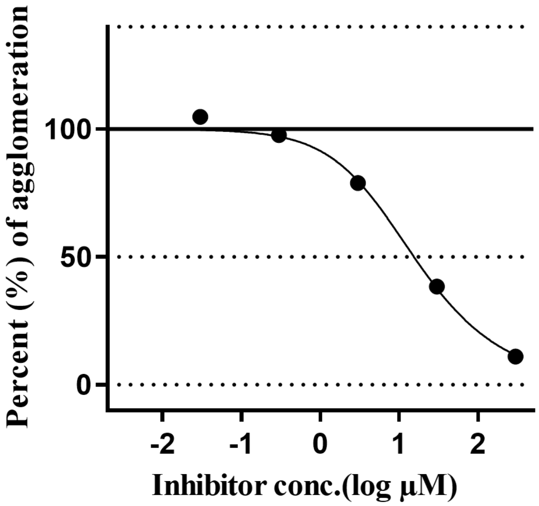

Figure S19. Inhibition of Aβ42 aggregation by rosmarinic acid.

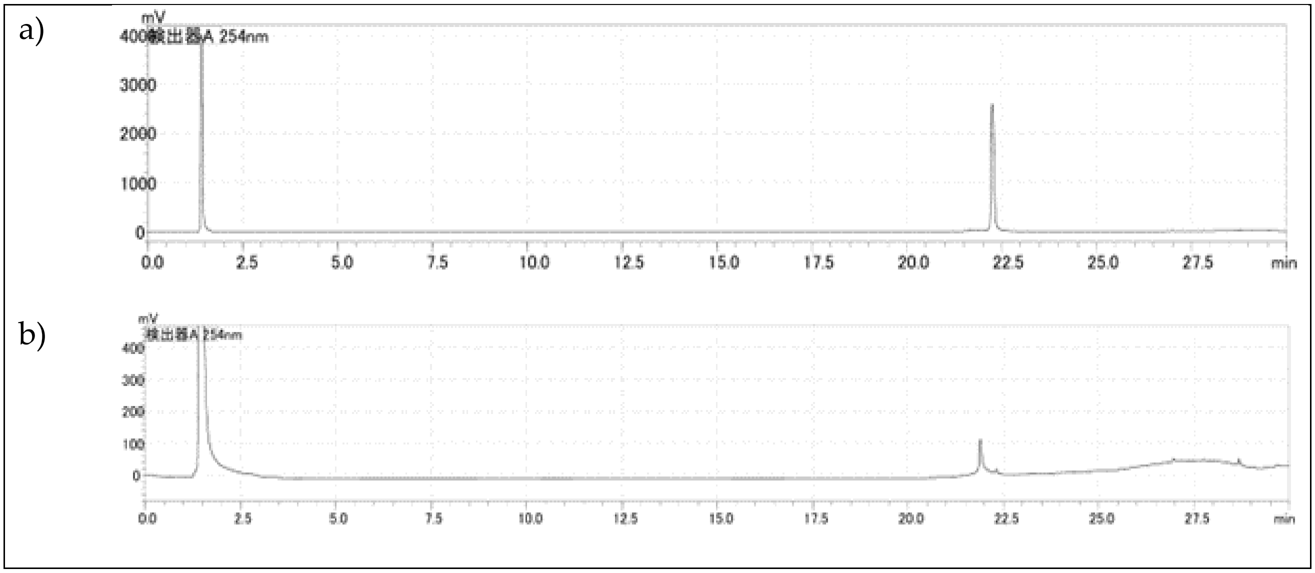

Figure S20. UV detection of PGG (a) and MG (b) in Liquid Chromatography.

The components were detected under UV using Shim-pack Velox C18 100Å 2.7 µm, 2.1×150 mm. The chromatographic run was started with two mobile phases consisted of 0.1% formic acid water (A) and 0.1% formic acid acetonitrile (B). Following gradient was applied at a flow rate of 200 µL/min: 0-2 min, 0% B; 2-16 min, 10% B; 16-22 min, from 40% B; 22-25 min, 100 0% B, column wash; 25-30 min, to 0% B for equilibration of the column. The sample concentration was 1 mg/mL and the injection volume was 10 µL. The detection wavelength was 254 nm.

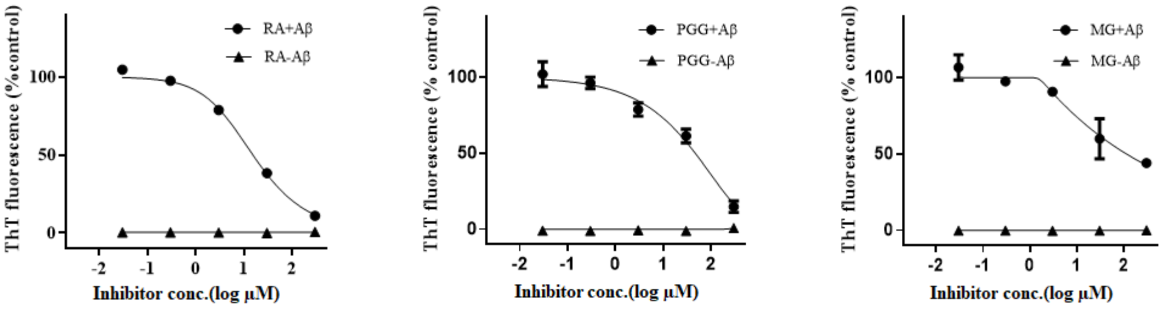

a) RA b) PGG c) MG

Figure S21. Effect of the compounds on ThT fluorescence in presence or absence of Aβ.
